# EMF 35 JMIP study for Japan’s long-term climate and energy policy: scenario designs and key findings

**DOI:** 10.1007/s11625-021-00913-2

**Published:** 2021-02-17

**Authors:** Masahiro Sugiyama, Shinichiro Fujimori, Kenichi Wada, Ken Oshiro, Etsushi Kato, Ryoichi Komiyama, Diego Silva Herran, Yuhji Matsuo, Hiroto Shiraki, Yiyi Ju

**Affiliations:** 1 Institute for Future Initiatives, The University of Tokyo, 7-3-1 Hongo, Bunkyo-ku, Tokyo, 113-0033 Japan; 2 Graduate School of Engineering, Kyoto University, Kyoto daigaku-katsura, Nishikyo-ku, Kyoto, 615-8530 Japan; 3 National Institute for Environmental Studies, 16-2 Onogawa, Tsukuba, Ibaraki 305-8506 Japan; 4 Research Institute of Innovative Technology for the Earth, 9-2 Kizugawadai, Kizugawa, Kyoto 619-0292 Japan; 5 Institute of Applied Energy, 1-14-2 Nishi-Shimbashi, Minato, Tokyo, 105-0003 Japan; 6 School of Engineering, The University of Tokyo, Hongo 7-3-1, Bunkyo-ku, Tokyo, 113-8656 Japan; 7 Institute for Global Environmental Strategies (IGES), 2108-11 Kamiyamaguchi, Hayama, Kanagawa, 240-0115 Japan; 8 Institute of Energy Economics, Japan, Kachidoki 1-chome, Chuo-ku, Tokyo, 104-0054 Japan; 9 The University of Shiga Prefecture, 2500, Hassaka-cho, Hikone-City, Shiga, 522-8533 Japan; 10 International Institute for Applied System Analysis (IIASA), Schlossplatz 1, 2361 Laxenburg, Austria

**Keywords:** Climate change mitigation, Integrated assessment, Long-term strategy, National climate policy, Uncertainty, Carbon neutrality, Net-zero emissions

## Abstract

In June, 2019, Japan submitted its mid-century strategy to the United Nations Framework Convention on Climate Change and pledged 80% emissions cuts by 2050. The strategy has not gone through a systematic analysis, however. The present study, Stanford Energy Modeling Forum (EMF) 35 Japan Model Intercomparison project (JMIP), employs five energy-economic and integrated assessment models to evaluate the nationally determined contribution and mid-century strategy of Japan. EMF 35 JMIP conducts a suite of sensitivity analyses on dimensions including emissions constraints, technology availability, and demand projections. The results confirm that Japan needs to deploy all of its mitigation strategies at a substantial scale, including energy efficiency, electricity decarbonization, and end-use electrification. Moreover, they suggest that with the absence of structural changes in the economy, heavy industries will be one of the hardest to decarbonize. Partitioning of the sum of squares based on a two-way analysis of variance (ANOVA) reconfirms that mitigation strategies, such as energy efficiency and electrification, are fairly robust across models and scenarios, but that the cost metrics are uncertain. There is a wide gap of policy strength and breadth between the current policy instruments and those suggested by the models. Japan should strengthen its climate action in all aspects of society and economy to achieve its long-term target.

**Supplementary Information:**

The online version contains supplementary material available at 10.1007/s11625-021-00913-2.

## Introduction

In accordance with Article 4 of the Paris Agreement, the Government of Japan submitted its long-term low greenhouse gas emission development strategy (or mid-century strategy) to the United Nations Framework Convention on Climate Change (UNFCCC) in June 2019 (Government of Japan 2019). The strategy mentioned Japan’s goal of reducing greenhouse gas emissions by 80% by 2050, which was stated in an earlier document (Ministry of the Environment 2012; Government of Japan 2016). Recently, in October 2020, Prime Minister Suga made a pledge to net-zero emissions by 2050 (Suga 2020). However, it is not immediately clear how Japan could achieve deep decarbonization while the consequences of and policy choices after the 2011 Fukushima nuclear disaster still linger, including difficulties of nuclear restarts and the rise of coal-fired power.

Although the Japanese government has not formally conducted a quantitative analysis of the proposal, many studies have already examined long-term policy proposals, including economy-wide climate policies (Fujimori et al. 2019; Kato and Kurosawa 2019; Oshiro et al. 2019; Sugiyama et al. 2019). Other studies have analyzed power sector policies that feature the significant penetration of variable renewable energies (VREs) (Komiyama et al. 2015; Matsuo et al. 2018).

While these studies have advanced our understanding of the policy issues, they have not comprehensively analyzed all the relevant factors. An important factor that has not received enough attention is the inter-model uncertainty among energy-economic and integrated assessment models, which is crucial in informing the climate policy debate (Krey 2014).

To address the issue of inter-model uncertainty, the Stanford Energy Modeling Forum 35 (EMF) Japan Model Intercomparison Project (JMIP) is tasked with analyzing Japan’s climate policy with a multi-model framework. The present study extends a pilot study by Sugiyama et al. (2019) and explores uncertainties in policy, technology, demand, and import dimensions in a systematic manner.

In particular, this study asks the following research questions:

- (1) How do various types of uncertainties affect the cost, feasibility, and features (e.g., power generation mix) of Japan’s mitigation policy?
- (2) Is there a specific, robust pattern in Japan's decarbonization pathways that cuts across uncertainties? What is the policy implication, given the magnitude of uncertainties?

Though our primary focus is on the 80% emissions reduction, we also discuss the implications for the net-zero target.

Some words on the definition of uncertainty are in order. There are many sources of uncertainties, including structural and parametric uncertainties. This paper classifies the source of uncertainties into those originating from scenario specification (inter-scenario uncertainty) and the remainder, model uncertainty, which encompasses both structural and unharmonized parametric uncertainties. This definition is methodological, not conceptual. This is also consistent with our statistical approach.

This paper lays out the scenario design and some key findings of the EMF 35 JMIP study. Detailed investigations into the role of variable renewables (Shiraki et al. 2021), end-use electrification (Sakamoto et al. 2021), and industrial mitigation (Ju et al. 2021) are presented in the companion papers in this special feature. They are further enriched by individual modeling papers in this special issue.

The rest of the paper is organized as follows. The “Policy and literature review” section presents a short summary of Japan’s climate policy and the modeling literature. Although our main focus is on modeling, we provide a fairly broad overview of Japan’s policy situation. The “Method” section describes the models used and the scenarios utilized. This is followed by the “Results” section, which presents the outcomes of the five-model analysis. The paper concludes with “Discussion and conclusions”.

## Policy and literature review

### Policy review

This section gives a brief overview of Japan’s climate policies and places the present analysis in a wider context, given that the policymaking in Japan is quite different from the western countries (Sofer 2016) in that Japan’s climate policy has been mostly shaped by bureaucracies, and other stakeholders played a limited role (Kameyama 2016). This section is based on earlier reviews by Takase and Suzuki (2011), Kuramochi (2015), and Kuriyama et al. (2019). To understand the political economy aspects, see Kameyama (2016), Sofer (2016), and Trencher et al. (2019) and the references therein. Kameyama (2016) chronicled the climate policy of Japan from 1980s until 2015, focusing on the role of premiership. Sofer (2016) gave a concise summary of the actors and their roles in Japan’s climate policy, contrasting Japan and the United States. Trencher et al. (2019) is centered around coal-fired power plants, for which Japan has been supporting domestic usage and exports. The review here focuses on the central government and does not cover sub-national or non-state actors.

Japan’s climate policy was based mainly on energy efficiency measures, such as Top-Runner Programs (Inoue and Matsumoto 2019) and building codes and labeling (Murakami et al. 2009; MLIT 2016), and voluntary actions taken by the industry (Keidanren 2013, 2019; Wakabayashi 2013; Wakabayashi and Arimura 2016). These are mainly under the remit of the Ministry of Economy, Trade, and Industry (METI). Though they are so called, voluntary action plans go through formal reviews by expert committees that are set up by the government. In particular, the Kyoto Protocol Target Achievement Plan formalized the review during the Protocol’s first commitment period. With regard to the promotion of lifestyle changes, the Ministry of the Environment has pushed for information campaigns, such as Cool Biz (since 2005). This campaign proved to be more extensive than its counterparts in other countries (Shove and Granier 2018).

Conversely, Japan has not been enthusiastic about price instruments. Overall, carbon pricing (both explicit and implicit) has been relatively weak in Japan (Ramstein et al. 2019). The fossil fuel tax, namely *chikyu ondanka taisaku zei* (tax for global warming countermeasures), stands at 289 JPY/t-CO_2_ or about 3 USD/t-CO_2_ (Ministry of the Environment 2020, partly because of a competitiveness concern for the industry. It is important to recognize that transport fuels have been taxed already at a high level. At the prefectural level, the Tokyo Metropolitan Government and Saitama Prefectural Government have been implementing an emissions trading scheme (ETS) for the commercial sector (Arimura and Abe 2020). The Tokyo ETS was successful during Phase 1 (2010–2014). A remarkable 25% reduction in carbon dioxide (CO_2_) emissions was partly attributable to the carbon price signal but also assisted by the energy savings after the 2011 energy crisis and the effect of an advisory system (Wakabayashi and Kimura 2018; Arimura and Abe 2020).

Currently, the electricity sector is going through rapid changes, including the retail deregulation of 2016, the unbundling of utilities in 2020, and new market frameworks (i.e., baseload, flexibility, non-fossil value, and capacity) (Hattori 2019). Compared to countries like Germany, Japan had a slow start in its transition to renewables (Cherp et al. 2017). The 2011 feed-in tariff (FIT) scheme helped in the growth of renewables. In particular, solar photovoltaics rose from 0.4% of Japan’s power generation in FY2011 to 6% in FY2018 (ANRE 2020a). However, the FIT also led to a gargantuan price tag of trillions of yen per year. The government is currently transitioning from the FIT scheme to a feed-in premium scheme and energy auctions to address the cost issue (Calculation Committee for Procurement Price, etc. 2020). Shiraki et al. (2021) in this issue reviews power sector policy development more fully.

However, Japan’s energy sector has not been fundamentally altered despite a series of reforms in energy policies after the 2011 nuclear disaster, because it is dictated by resource constraints and broader economic conditions. Japan has a relatively small renewable resource base compared to its electricity demand (Luderer et al. 2017) because of its high population density, and the costs of renewables are higher than those in other countries (IRENA 2019; Calculation Committee for Procurement Price, etc. 2020). Unlike many of Western countries, Japan retains a large presence of heavy industry. However, as the industry sector is one of hardest to decarbonize (Davis et al. 2018; Luderer et al. 2018) and innovative technologies have not been developed sufficiently (Ju et al. 2021), industrial mitigation presents a significant challenge for Japan.

### Quantitative policy targets

In the first commitment period of the Kyoto Protocol (2008–2012), Japan honored its commitment to reduce emissions by 6% from the 1990 levels by reducing domestic emissions and purchasing credits from abroad (Ministry of the Environment 2014). In June 2009, the Aso administration announced a mid-term target of 15% emissions reduction by 2020 relative to the 2005 levels (8% reduction relative to the 1990 levels) (Prime Minister’s Office 2009). A significant modeling exercise (as part of a policy process) was conducted in preparation for this target (Fukui 2009). In September 2009, however, the newly elected, Hatoyama administration of the Democratic Party of Japan (DPJ) announced its ambition to reduce its emissions by 25% by 2020 relative to the 1990 levels (33% reduction relative to the 2005 levels) (Copenhagen Pledge), but this plan required a significant expansion of nuclear power fleets (Duffield and Woodall 2011). The pledge was overturned after the 2011 Great Eastern Japan Earthquake, tsunamis, and the Fukushima Daiichi nuclear disaster. The DPJ contemplated an alternative energy path without relying on nuclear power. However, it lost to a coalition of the Liberal Democratic Party and Komeito in the 2012 election. Japan did not take part in the second commitment period of the Kyoto Protocol. Furthermore, it downgraded its 2020 pledge to 3.8% emissions reduction relative to the 2005 levels under the prospect of limited nuclear operation (Warsaw Target) (Ministry of the Environment 2013).

In the run-up to the COP21 in Paris, the Abe administration, which won the 2012 election, submitted its Intended Nationally Determined Contribution to the UNFCCC. Herein, Japan committed to reduce its emissions by 26% by FY2030^1^ from the FY 2013 levels (Government of Japan 2015). In the following year, the Cabinet approved the Plan for Global Warming Countermeasure, which included a goal to reduce emissions by 80% by 2050 (Government of Japan 2016). In 2019, the Government of Japan (2019) decided on its mid-century strategy and reiterated the 80% emissions reduction goal. In March 2020, in the 5-year update cycle of mitigation policies, Japan retained the formerly announced targets (Government of Japan 2020). Most recently, in October 2020, Prime Minster Suga made a pledge of net-zero emissions by 2050 in his inaugural speech in the parliament.

**Fig. 1:**
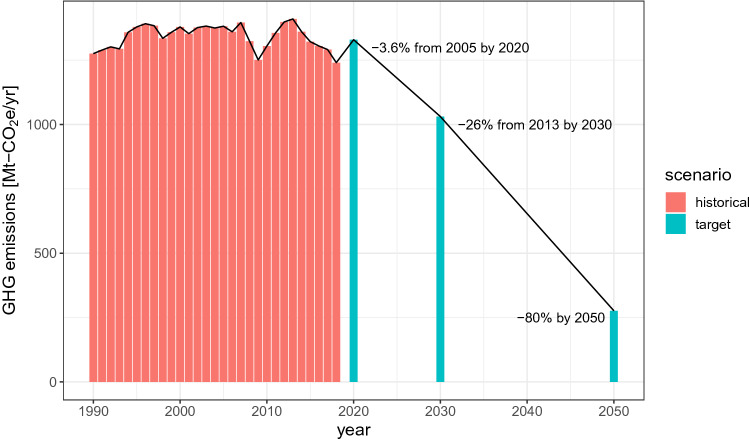
Historical GHG emissions, and 2020, 2030, and 2050 targets. Data are from (UNFCCC 2020). Note that the 2020 target is based on a strong assumption of no mitigation contribution from nuclear power

One topic of contention in Japan’s target is the choice of the reference year (Kuramochi 2015). The most significant is with respect to the Warsaw target such that a 3.8% reduction from the 2005 levels translates into a 3.1% increase from the 1990 levels. The reference year for the mid-century strategy had not yet been decided; this no longer matters since the government pledged a net-zero target (Fig. 1).

**Fig. 2:**
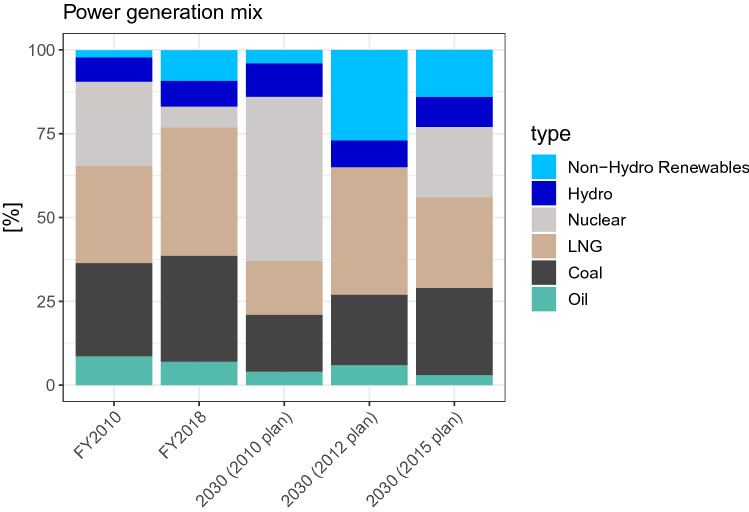
Power generation mix for FY2010 and FY2018 (actual), the 2030 target plans according to the 2010 (ANRE 2010), 2012 (Energy and Environmental Council 2012) and 2015 (METI 2015) plans. The 2030 (FY2010) plan corresponds to the *Saidai Dounyu* (maximum deployment) case. The 2030 (2012 plan) is from the nuclear-zero case

Another key feature of Japan’s long-term policy is that it is associated with a detailed emissions sectoral breakdown and energy mix (Fig. 2). Moreover, these numbers are not merely indicative targets but serve as concrete goals in policy discussions. For instance, under the nationally determined contribution (NDC), 22–24% of electricity is to be supplied by renewables, and there is an additional detailed breakdown for individual renewable technologies. Another contentious issue is the role of nuclear power, which is assumed to account for 20–22%. Although restarting nuclear power plants has been slow and only six units are operational as of April 20, 2020 (ANRE 2020b), the detailed breakdown of the power generation mix has not been revised during the update of the Strategic Energy Plan in 2018 (ANRE 2018). There are high expectations for an improvement in energy intensity of GDP with an annual improvement rate of 2.1% per year for 2014–2030, although the observed rate was 1.6% per year for 2000–2015. This could be the result of a high growth projection of gross domestic product (GDP), however (Kuriyama et al. 2019).

### Mid-century strategy

In contrast to the 2030 target, Japan’s 2050 policy document is vague with respect to numerous concrete issues (Government of Japan 2019). For instance, it does not specify the reference year or demonstrate any specific pathway to achieve the 80% emission reduction goal. Nonetheless, it mentions certain notable points. The Fifth Strategic Energy Plan (ANRE 2018) also provides useful information.

First, the long-term strategy and the Strategic Energy Plan states “multi-track scenarios” or pluralistic perspectives on scenarios, and in particular, technology development. This approach is in contrast to the Japanese approach with respect to the 2030 target, for which the government has allocated emissions reduction to each technology. Second, both documents place significant emphasis on the role of technological innovations in achieving the long-term goal, with the long-term strategy touting a virtuous cycle between economic growth and mitigation. Furthermore, it mentions the link with related innovation strategies the government has already formulated. Lastly, the Strategic Energy Plan proposes a scientific review mechanism through which the government periodically reviews progress toward the transition to a clean energy system. This point has not been emphasized in the long-term strategy. It is not clear how modeling studies, such as the present one, could contribute to this proposed review mechanism.

### Modeling: single-model studies

Many studies have focused on economy-wide, long-term climate change mitigation for Japan up to 2050. These can be classified into (1) single-model studies and (2) multi-model studies. For sectoral-level reviews, please refer to the companion papers (Ju et al. 2021; Sakamoto et al. 2021; Shiraki et al. 2021).

For single-model studies, Kainuma et al. (2015) used the AIM/Enduse energy systems model to analyze the implications of 80% emissions reduction by 2050. Oshiro et al. (2018) employed AIM/Enduse to analyze net zero emissions of CO_2_ by 2050, and found the importance of bioenergy with carbon capture and storage (BECCS). In a similar vein, Kato and Kurosawa (2019) examined 2050 emissions reduction of 80% and more, and found that reduced service demands and the availability of BECCS would be vital to achieve 90% emissions reduction. Schreyer et al. (2020) used the ReMIND model to compare 2050 net-zero targets for Australia, the European Union, Japan, and the United States, and found a smaller share of variable renewables in Japan because of its high population density.

### Modeling: multi-model studies

Among multi-model studies in Japan, the earlier ones were part of the government-led policy process. In recent years, we have seen an increasing number of academic studies, including our pilot phase research (Sugiyama et al. 2019).

Government-led efforts include the Mid-Term Target Evaluation Committee (*Chuki Mokuhyo Kento Iinkai*) (Fukui 2009) and the Energy and Environmental Council (2012) (*Enerugi Kankyo Kaigi*). Both exercises were conducted as part of the policymaking process with town hall meetings and deliberative polls. They mainly analyzed six and three scenarios, respectively. The former analyzed different emissions reduction levels and policy packages, and the (modified) middle option out of the six was eventually chosen. The latter focused on different levels of nuclear power generation, and the zero nuclear case was finally selected. Unfortunately, these model inter-comparison results were not published in the academic literature, unlike the EMF studies in the United States (Fawcett et al. 2014) or Europe (Knopf et al. 2013).

In the academic literature, one of the recurring themes is the high marginal abatement costs in Japan. A five-model study by Hanaoka and Kainuma (2012) examined medium-term (2020 and 2030) marginal costs of abatement but did not focus on emissions pathways. The Asian Modeling Exercise (AME) (Calvin et al. 2012) implemented scenarios of idealized carbon prices and globally coordinated scenarios, in which four models from Japan participated. Aldy et al. (2016) contrasted the marginal cost of Japan against those from other parts of the world. Our pilot study (Sugiyama et al. 2019) compared the cost of 80% emissions reduction by 2050 in Japan against those in the United States and Europe. These four studies revealed that the marginal cost in Japan is higher than that in other countries.

As part of the EU-funded MILES project, Akimoto et al. (2015) used DNE21 + and AIM/Enduse models to analyze the intended NDC of Japan. For the EU-funded CD-Links project, Oshiro et al. (2019) compared global IAM results against two, national models (AIM/Enduse [Japan] and DNE21 + (national)), and demonstrated that Japan’s goal of 80% emissions reduction is consistent with cost-effective pathways for the 2-degree target, but not with the 1.5-degree target.

Although these studies are of crucial importance, they do not fully characterize the inter-model uncertainty in assessing the 2050 target, including technology availability (Clarke et al. 2014a). For instance, in the wake of the Fukushima nuclear disaster, more attention has been paid to the future of power generation mix, and the costs of bringing about a desired mix. And yet, it is well known (at least at the global scale) that such a power mix is subject to enormous uncertainty.

Moreover, the inter-model uncertainty interacts with other sources of uncertainty. Sugiyama et al. (2019) conducted an initial assessment of inter-model uncertainty, but did not fully consider other types of uncertainty, including policy stringency, technological availability, service demand reduction, and import prices. To address these issues, the present study conducts a multi-model assessment of Japan’s long-term climate policy under varying future scenarios.

## Method

### Models

Five energy-economic and integrated assessment models are used in the present study: AIM/Hub-Japan, AIM/Enduse-Japan, DNE21, IEEJ_Japan 2017, and TIMES-Japan. (DNE21 should not be taken for DNE21 +, which is a different model.) These differ in model type, regional aggregation level and technological representation. As shown below, using a variety of models leads to a wide range of assessment results, confirming the usefulness of the analysis of inter-model uncertainty.

Table 1 shows the summary of models used in the present study. A detailed description of each model can be found in the Electronic Supplementary Material (ESM) (“Model descriptions”).

**Table 1:** Participating energy-economic and integrated assessment models to assess the climate policies in Japan

Model	Coverage	Institute	Model type	Representative reference (see ESM for fuller descriptions)
AIM/Enduse-Japan V2.1	National	Kyoto University and National Institute for Environmental Studies (NIES, Japan)	Recursive dynamic, partial equilibrium	Oshiro and Masui (2015)
AIM/Hub-Japan 2.1	National	Kyoto University, National Institute for Environmental Studies (NIES, Japan) and Institute for Global Environmental Strategies (IGES)	Recursive dynamic, general equilibrium	Fujimori et al. (2017)
DNE21 Version 1.3	Global	The University of Tokyo (UTokyo)	Perfect foresight, partial equilibrium	Fujii et al. (2015)
IEEJ Japan ver. 2017	National	Institute of Energy Economics, Japan (IEEJ)	Perfect foresight, partial equilibrium	Matsuo et al. (2013)
TIMES-Japan 3.1	National	The Institute of Applied Energy (IAE), Japan	Perfect foresight, partial equilibrium	Kato and Kurosawa (2019)

AIM/Hub-Japan is a computable general equilibrium model while AIM/Enduse-Japan is a bottom-up, technology-rich model

Some models cover multiple greenhouse gases, but this study focuses on CO_2_ emissions from energy use and industrial processes.

### Scenarios

The scenario design of this study examines four dimensions of uncertainty (Table 2):

- emissions constraint stringency;
- technological sensitivity;
- service demand levels; and
- energy import prices.

**Table 2:** Description of EMF 35 JMIP scenarios

Dimension	Scenarios	Notes
Policy stringency (emissions constraint)	26by30 + 80by50_Def 26by30 + 70by50_Def 26by30 + 90by50_Def 26by30 + 100by50_Def 16by30 + 80by50_Def 36by30 + 80by50_Def	NDC and mid-century strategy NDC and 70% reduction by 2050 NDC and 90% reduction by 2050 NDC and 100% reduction by 2050 16% reduction by 2030 and mid-century strategy 36% reduction by 2030 and mid-century strategy
Technology sensitivity	26by30 + 80by50_NoCCS 26by30 + 80by50_LimNuc 26by30 + 80by50_NoNuc 26by30 + 80by50_HighInt 26by30 + 80by50_LoInt 26by30 + 80by50_LoVREcost 26by30 + 80by50_HiVREcost 26by30 + 80by50_LoVREpot 26by30 + 80by50_HiVREpot 26by30 + 80by50_LoStorageCost	No carbon capture and storage (CCS) is available Only limited deployment of nuclear is allowed Nuclear power is not available High challenges of renewables system integration Low challenges of renewables system integration The costs of renewables are halved The costs of renewables are doubled The potentials of renewables are halved The potentials of renewables are doubled The cost of energy storage is greatly reduced
Service demand levels	26by30 + 80by50_LoDem 26by30 + 80by50_LoDemBld 26by30 + 80by50_LoDemTra 26by30 + 80by50_LoDemInd	A lower GDP scenario is applied Lower GDP and demands halved for buildings Lower GDP and demands halved for transport Lower GDP and demands halved for industry
Energy import prices	26by30 + 80by50_HiImportCost	Energy import prices are doubled

Only policy scenarios are shown for brevity. Note that baseline scenarios are denoted as Baseline_Def, etc. See the ESM *Scenario Descriptions* for more details

There are some differences in the implementation of scenarios in each model. For instance, for the LoVREcost scenario, some models implemented the VRE cost reduction from the beginning of the calculation period while others reduced the cost in a linear schedule

The detailed scenario descriptions are given in the ESM (“Scenario descriptions”). Unlike previous EMF studies (e.g., EMF 27) (Kriegler et al. 2014), we did not combine variations in different dimensions to produce a scenario matrix since in our case, the number of scenarios would have been prohibitively large.

The name of each scenario is denoted as (*policy dimension*)_(*other parameter settings*). (*policy dimension*) takes the format of either “Baseline” or “(xx)by30 + (yy)by50”, which stipulates xx% reduction by 2030 and yy% reduction by 2050. The main scenarios of our study are as follows:

- Baseline_Def: no climate policy^2^ assumed with default parameter settings:
- 26by30 + 80by50_Def: each model imposes Japan’s NDC (26% emissions reduction by FY2030 relative to the FY2013 levels) and mid-century strategy (80% emissions reduction by 2050).

The different levels of emission constraints are analyzed to explore the implications of the over- and under-achievement of current policies. This is also useful to inform the ratchet-up mechanism in the Paris Agreement, though the Government of Japan has already submitted its updated NDC in March without revising its goal for 2030 (Government of Japan 2020).

The technology sensitivity analysis follows previous EMF studies (Knopf et al. 2013; Clarke et al. 2014a; Fawcett et al. 2014) and analyzes the impacts of the availability of various technological options in an idealized manner. In addition, this study looks at renewables and systems integration (including energy storage). As nuclear power is such a divisive issue, we consider three nuclear scenarios: model default, limited nuclear, and no nuclear. Availability of a technological option is affected by technological development, public acceptance, or both.

Energy service demands are an important factor in determining the mitigation challenges (Fujimori et al. 2014; Grubler et al. 2018; Kuriyama et al. 2019). Our scenario design includes idealized sensitivity analyses to reduce the service demands by half in each of the three sectors (industry, transport, and buildings), besides a scenario with lower economic growth rate. Although we treat them as idealized scenarios, a myriad of factors can induce changes in service demands, including a sudden demand shock, such as the 2019–2021 outbreak of the novel coronavirus and improvements in material efficiency.

Japan relies heavily on energy imports with a self-sufficiency rate of less than 10% (ANRE 2019). Even after transitioning to a clean energy system, Japan may continue to rely on imports. Currently the government is exploring the possibility of importing a significant amount of hydrogen (Ministerial Council on Renewable Energy, Hydrogen and Related Issues 2017) from countries, such as Australia (Ozawa et al. 2017). It is therefore useful to examine the sensitivity to energy import price changes.

### Harmonization of GDP and population

In previous EMF studies, it was a standard practice to not harmonize basic input assumptions. While this approach is useful in characterizing variations in such parameters, an alternative strategy involves harmonizing basic inputs so that the analysis can focus on model structures and more detailed technical parameters. In this study, we harmonize gross domestic product (GDP) and population, two key drivers of energy consumption and greenhouse gas (GHG) emissions.

Population data were adopted from (IPSS 2017). We assume two GDP growth scenarios. The high growth scenario uses data on the growth rate till 2030 from the government’s Long-Term Energy Outlook, and selects the 2030–2050 growth rates, from the Shared Socioeconomic Pathway (SSP) 2 (Dellink et al. 2017). The low growth scenario presumes the SSP 2 growth rate throughout. Scenarios with “LoDem”, “LoDemInd”, “LoDemBld”, and “LoDemTra” also have a low GDP growth rate. Although we consider only one population scenario and two GDP scenarios, service demand sensitivity scenarios provide an opportunity to explore the impact of drivers in an idealized manner. Further details are provided in the ESM *Scenario Descriptions*. The scenario submission status is summarized in Table ESM 4.

### Decomposition of variance (sum of squares)

Our rich dataset is underlined by five models and 38 scenario settings. To identify robust areas and uncertain domains, we compare the variance of the normalized value of each variable and decompose the variance.

Specifically, we partition the sum of squares of a two-way analysis of variance (ANOVA) model (NIST/SEMATECH 2013; Takakura et al. 2019):

1 $$y_{{m,s}}  = \mu  + \alpha _{{m~}}  + \beta _{s}  + \gamma _{{m,s~}}  + \epsilon _{{m,s}} ,$$

where *y* is a generic, *normalized* model variable for a certain period, the subscripts *s,* and *m* denote scenarios and models, respectively. $\mu$ is the mean response. $\alpha _{m}$ and $\beta _{s}$ represent the main effect of model and scenario, respectively. $\gamma _{{m,s}}$ is the interaction term, and $\epsilon _{{m,s}}$ is the residual term. To compare across variables, we restrict ourselves to mitigation scenarios with the NDC and mid-century strategy (scenario name starting with 26by30 + 80by50), and normalize all variables by its mean across scenarios and models.

The sum of squares can be decomposed as

2 $$ {\text{SS}}_{{{\text{total}}}}  = {\text{SS}}_{m}  + {\text{SS}}_{s}  + {\text{SS}}_{i} , $$

where SS_total_ is the total sum of squares $\sum\nolimits_{{m,s}} {(y_{{m,s}}  - \bar{y})^{2} }$, with the bar denoting the pooled mean. SS_m_, SS_s_, and SS_i_ represent the sums of squares attributable to models, scenarios, and interactions, respectively.

## Results

First, we focus on selected scenarios (emissions constraints of the NDC and mid-century strategy) to highlight key features and explore the parameter sensitivities of no nuclear power, no carbon capture and storage (CCS), and lower GDP growth. The choice of this set is motivated by the following considerations. First, nuclear power remains a contentious political issue. Second, CCS is often considered to be a key enabler of deep decarbonization (Kriegler et al. 2014; Clarke et al. 2014a). Third, there is criticism against the government projection of GDP (Kuriyama et al. 2019). As shown below, these factors have a large impact on policy costs.

**Fig. 3:**
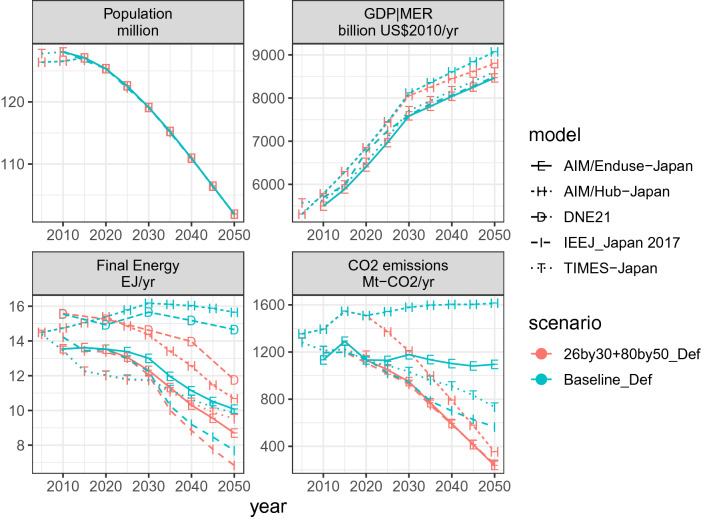
Drivers [population (upper left) and economic growth (upper right)], final energy (lower left), and CO_2_ emissions (lower right) from energy and industrial processes. Note that AIM/Hub-Japan calculates GDP endogenously

Figure 3 presents the time series of the two key drivers (population and gross domestic product or GDP), total final energy consumption, and CO_2_ emissions from energy use and industrial processes for the baseline and NDC and mid-century strategy scenario (for other scenarios, see Fig. ESM 1). Although the population is projected to decrease by 19% from 2020 to 2050, the Japanese economy is assumed to grow by approximately 30% over the same timeframe. There is a significant variation in final energy and emissions in the baseline scenario, which reconfirms the need for model inter-comparison. The IEEJ_Japan 2017 model shows a baseline emissions trajectory that is similar to the policy case (26by30 + 80by50_Def) because of assumed energy efficiency trends. Emissions in the base year from AIM/Hub-Japan are different from those of other models because of the use of a different database (see the ESM section *Energy data sources and model treatment*).

**Fig. 4:**
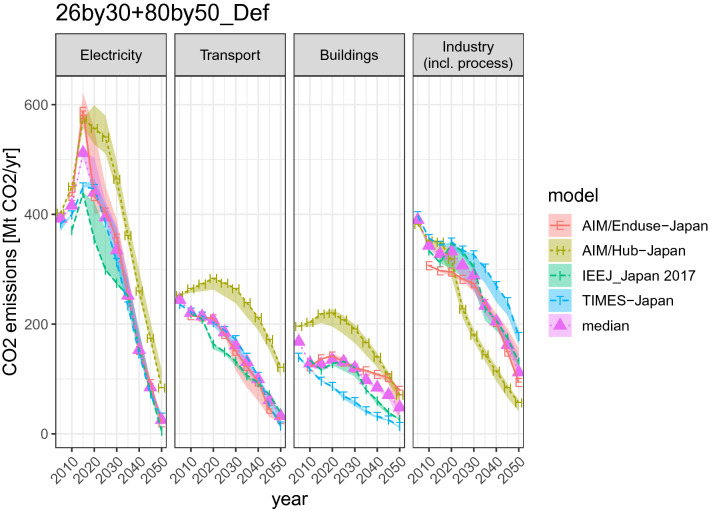
Sectoral CO_2_ emissions for the selected scenarios. The lines correspond to the 26by30 + 80by50_Def scenario. The ribbons represent the range of NoNuc, NoCCS, LoDem, and Def scenarios (the scenario prefix “26by30 + 80by50_” is dropped)

Figure 4 disaggregates emissions reduction into different sectors, thereby demonstrating how Japan can reduce its own emissions by 2050. There is a difference between the partial equilibrium and general equilibrium models. The former chooses almost complete decarbonization of the power and transport sectors by 2050, whereas there are some differences in the buildings sector. The industry emission is the most difficult to abate, as shown in our previous research (Sugiyama et al. 2019). On the other hand, AIM/Hub-Japan, the only general equilibrium model, exhibits a significant emissions reduction for industry. In AIM/Hub-Japan, the hardest sector to decarbonize is transportation. Figure 4 also displays the model range of emissions across scenarios, represented by ribbons. The cross-scenario range is dominated by the inter-model differences.

**Fig. 5:**
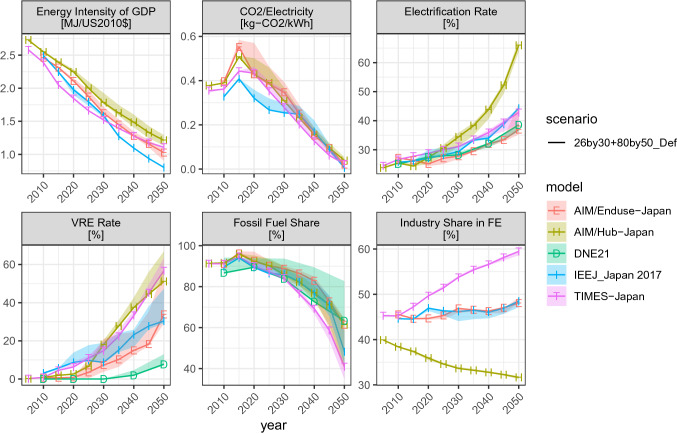
Key indicators of decarbonization options: (top left) energy intensity of GDP, (top middle) CO_2_ intensity of electricity, (top right) the share of electricity in final energy consumption, (bottom left) the share of solar and wind in secondary electricity, (bottom middle) share of fossil fuels in primary energy, and (bottom right) the share of the industry sector in total final energy consumption. The ribbons represent the ranges of NoNuc, NoCCS, LoDem, and Def scenarios (the scenario prefix “26by30 + 80by50_” is dropped).

To understand the type of approaches used by models to achieve deep emissions cuts, Fig. 5 characterizes the key indicators of mitigation for the four main scenarios, with 26by30 + 80by50_Def represented by solid lines and other scenarios depicted by ribbons. The figure reveals that the options that are found to be useful in the global context are also effective in Japan: economy-wide energy efficiency (Clarke et al. 2014b; Sugiyama et al. 2014), power sector decarbonization (Clarke et al. 2014b; Krey et al. 2014), end-use electrification (Williams et al. 2012; Sugiyama 2012; Krey et al. 2014), penetration of VREs (Luderer et al. 2014), and a shift away from fossil fuels (Krey et al. 2014; IPCC 2018). Robustness varies by indicator. Energy efficiency and electricity decarbonization are most robust, and the electrification rate changes by model. The increasing tendencies of VREs and non-fossil energy are robust but the magnitudes are uncertain. The share of industry in final energy consumption increases with time in the partial equilibrium models, a tendency consistent with Fig. 4.

Our focus is on the mid-century strategy (80% emissions reduction), but we find that the same strategies are also effective in more stringent cases, though they are further strengthened (Fig. ESM 11). Note that the 26by30 + 90by50_Def scenario is infeasible in two models, and the 26by30 + 100by50_Def in three models (Table ESM 4).

For electrification, AIM/Hub-Japan shows a higher rate than other models. The reason for this is due to high electrification of the industry sector (Fig. ESM 2) (see Sakamoto et al. 2021 for more on this). Also, the industry share of final energy decreases in AIM/Hub-Japan not because the industry final energy decreases more rapidly than in other models, but because the total final energy consumption does not reduce as much as other partial equilibrium models (Fig. ESM 3).

On the basis of per-capita indicators, the median final energy consumption decreases by 11% from 2010 to 2050, while the median value of electricity consumption increases by 43% (see Figures ESM 4 and 5).

There are some variations across scenarios in the share of VREs and fossil fuel shares, but they are not as large as the inter-model uncertainties. A large fossil fuel share found for DNE21 is from the NoNuc scenario, in which the model prefers natural gas power plants with CCS (Fig. 7).

Another uncertain variable is the use of CCS. The median CCS sequestration is about 50 Mt-CO_2_/year in 2050, with the maximum amount being approximately 350Mt-CO_2_/year for AIM/Hub-Japan (Fig. ESM 5).

There is a discrepancy in the industry share of final energy consumption even in the base year. This is attributed to the difference in the database used among the participating models. The models use either the energy balance of the International Energy Agency or the comprehensive energy statistics compiled by METI. There are some differences between these two databases, and the variations are pronounced for the industry share (Aoshima 2008).^3^ See the ESM *Energy data sources and model treatment* for a fuller description.

**Fig. 6:**
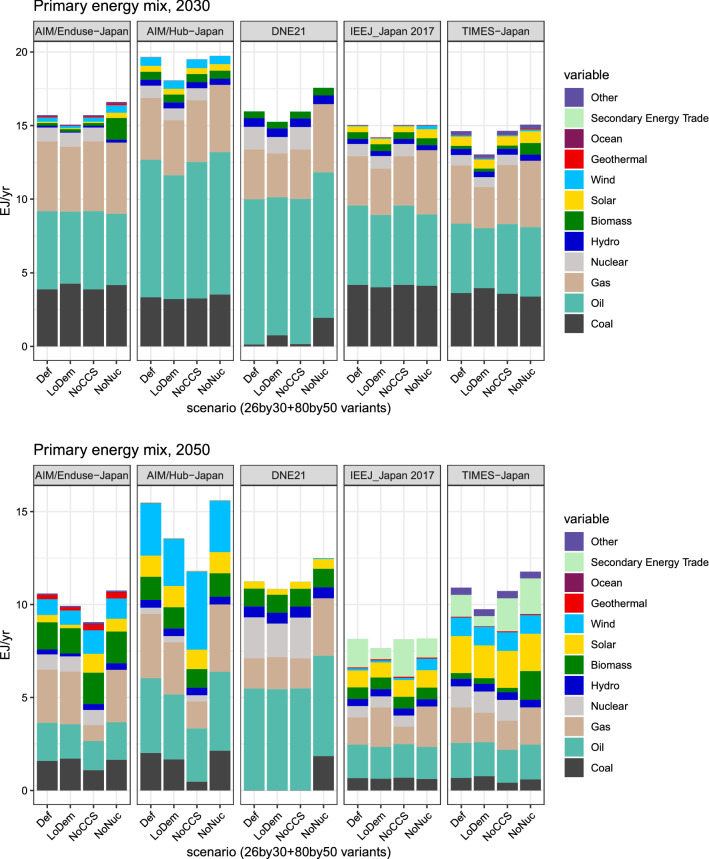
Primary energy mix for the selected scenarios for 2030 (top) and 2050 (bottom)

**Fig. 7:**
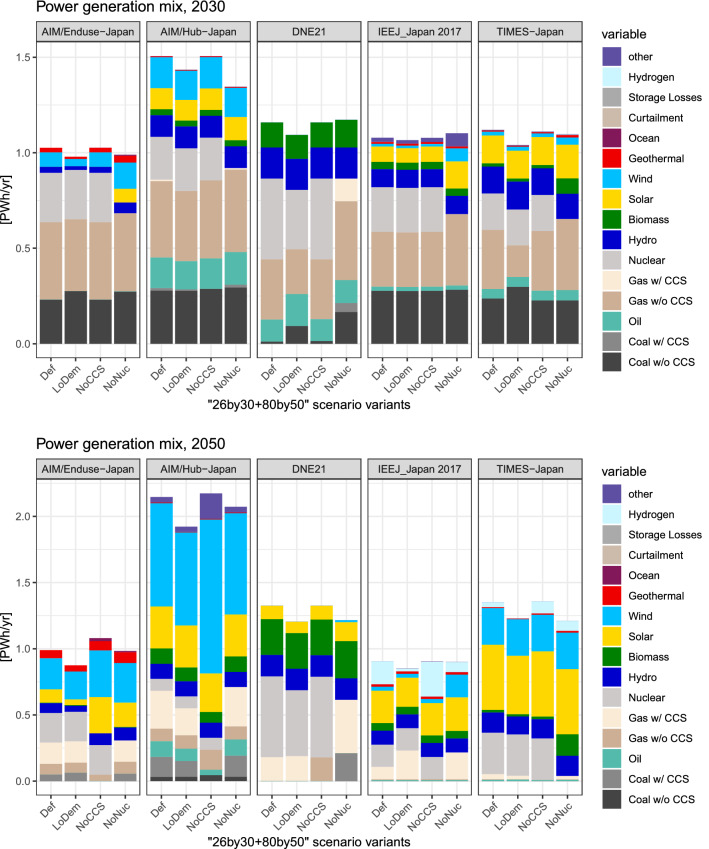
Power generation mix in 2030 and 2050 for the selected scenarios. The “other” in AIM/Hub-Japan refers to power generation technologies, such as ocean, tidal, etc.

Figures 6 and 7 describe the primary energy and power generation mixes for different scenarios for 2030 and 2050. The ESM presents the compositions of energy and power generation in the baseline scenario, which are dominated by fossil fuels (Fig. ESM 7 for 2010; Figs. ESM 8 and 9 for 2030 and 2050, respectively). The penetration of renewables is limited in the baseline scenario partly because of high costs.

In 2030, fossil fuels are still dominant, and clean energy sources greatly expand after then (Fig. 6). In 2050, the models exhibit differing primary energy supply levels. It is 8EJ/year for IEEJ_Japan 2017 and 16EJ/year for AIM/Hub-Japan. They also show different preferred mixes, with their mixes strongly reflecting model defaults, despite scenario influences. In the primary energy mix, oil and gas (often with CCS) continue to play an important role for all the models even in 2050, irrespective of scenarios. The secondary energy trade, which represents hydrogen imports, is projected to play an increasing role in IEEJ_Japan 2017 and TIMES-Japan. Note that both models incorporate domestic hydrogen production and imports, and that imports predominate because of cost considerations and renewable resource limitations for green hydrogen (see Sakamoto et al. 2021 for more on this).

Power sector decarbonization accelerates significantly after 2030 (Fig. 7). The 2030 power generation mix should be compared with the official targets (Fig. 2) that fixes the share of nuclear power at around 20%. By design, our analysis considers a scenario without nuclear power, and the results include a power mix that is quite different from the official target.

As with total primary energy, total power generation varies greatly across models. In 2050, it ranges from 0.9 PWh/year in IEEJ_Japan 2017 to approximately 2.1 PWh/year in AIM/Hub-Japan. VREs expand greatly, with a median penetration rate of 42% among the four models (AIM/Hub-Japan, AIM/Enduse-Japan, IEEJ_Japan 2017, and TIMES-Japan). The exception to this is DNE21, which prefers nuclear power (Shiraki et al. 2021). When CCS or nuclear power is not available, the gap is compensated for by other clean energy sources, but different models exhibit different preferred generation methods. For instance, in IEEJ_Japan 2017, the unavailability of nuclear power increases gas with CCS and wind, and hydrogen increases when CCS is not available. Nuclear power is replaced with biopower in TIMES-Japan, and the unavailability of CCS increases hydrogen. A large deployment of wind in AIM/Enduse-Japan and AIM/Hub-Japan can be explained by larger wind resource potentials in these models (Shiraki et al. 2021).

**Fig. 8:**
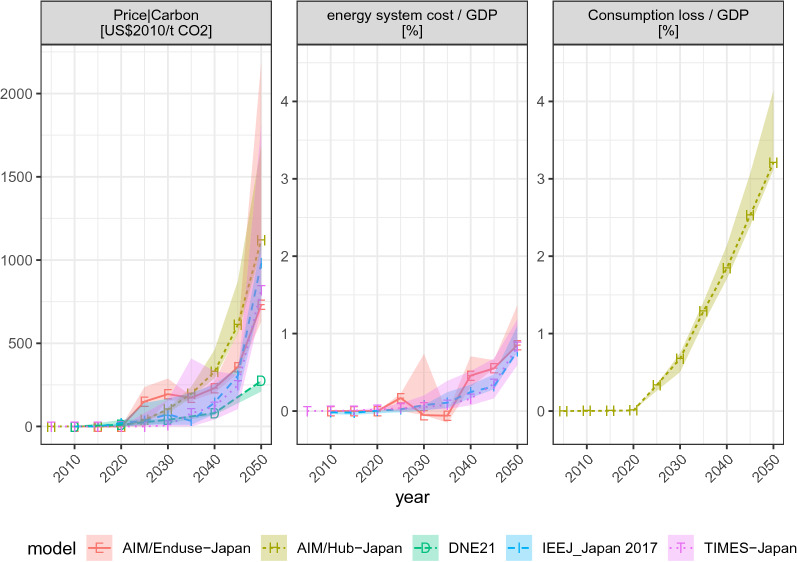
Marginal cost and policy costs (energy system cost and consumption loss) for the selected mitigation scenarios. The ribbons correspond to the uncertainty range represented by the four scenarios of the 26by30 + 80by50 scenario variants: Def, NoNuc, NoCCS, and LoDem

Next, we characterize the costs of achieving deep emissions reduction (Fig. 8) by examining marginal costs and total costs (consumption loss for AIM/Hub-Japan and additional total energy system cost for other bottom–up models). The carbon prices rise exponentially with time. The median price (2010USD/t-CO_2_) is 0 in 2020, 74 in 2030, 144 in 2040, and 819 in 2050 for the main mitigation scenario (26by30 + 80by50_Def). In the case of the 26by30 + 80by50_LoDem scenario, the median price is 0 in 2020, 18 in 2030, 75 in 2040, and 709 in 2050.

The values are sensitive to scenario assumptions. Though model fingerprints persist, the unavailability of CCS increases the marginal cost of mitigation in many models (Fig. ESM 10), leading to a wide range of uncertainty, as represented by ribbons. Total cost metrics are less sensitive. In 2050, the policy costs amount to approximately 3% of GDP for AIM/Hub-Japan, while other partial equilibrium models suggest 0.8–0.9% of GDP.

**Fig. 9:**
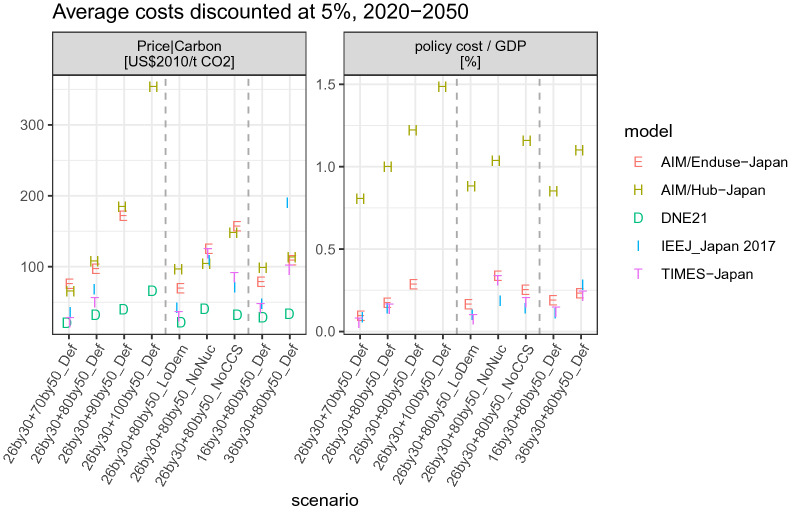
Sensitivity of average cost metrics (discounted at 5%, over 2020–2050) to scenario assumptions. Carbon price (left) and policy cost per GDP (right). Policy cost/GDP is defined as consumption loss per GDP loss for AIM/Hub-Japan and the additional total energy system cost per GDP for other models

To compare the cost metrics in a more concise manner, Fig. 9 presents the average costs (both total and marginal) discounted at 5% for the period 2020–2050. The two most stringent scenarios (90% or 100% emissions reduction) are feasible only for AIM/Hub-Japan and DNE21. Total costs roughly scale linearly with stringency, whereas marginal costs increase exponentially. The inter-model uncertainty range is sizable for both metrics, but particularly large for marginal costs.

Sensitivity analysis of the parameter setting reveals that lower demand and availability of nuclear power and CCS aid in containing the costs. In terms of policy costs, as compared to CCS, nuclear power has a larger impact in all the models, except AIM/Hub-Japan. For marginal costs, AIM/Hub-Japan and AIM/Enduse-Japan suggest lower impacts due to the lack of nuclear power than CCS; the rest of the models point in a different direction.

We also examine the impacts of setting different 2030 targets. Imposing a stricter target leads to higher costs in all the models, but AIM/Hub-Japan shows a nuanced behavior. In fact, the difference in the discounted carbon price between the 26by30 + 80by50_Def (99 2010USD/t-CO_2_) and 36by30 + 80by50_Def (105 USD-tCO_2_) scenarios is small. This is because early action leads to a higher cost in an earlier period but a lower cost in later periods. As the AIM/Hub-Japan is a myopic model, an early mitigation action partially improves welfare in their modeling framework.

**Fig. 10:**
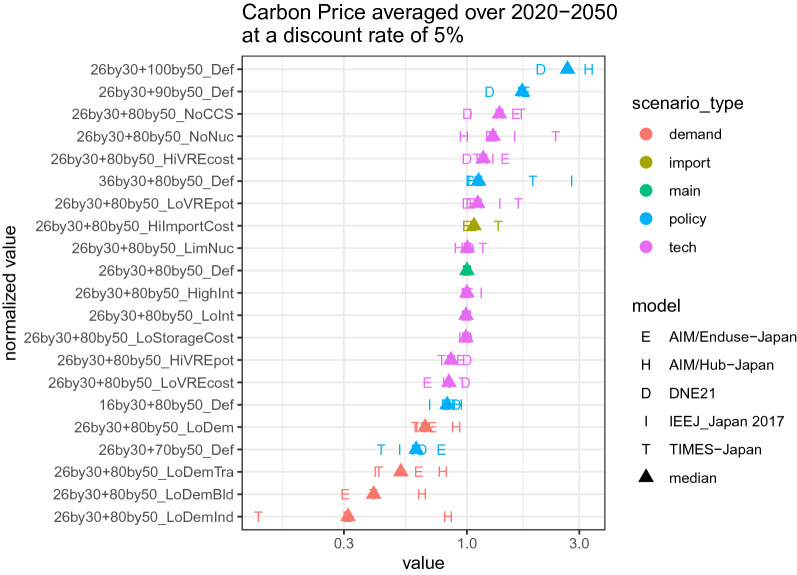
Discounted averages of the normalized carbon price in each scenario. Discounting is over 2020–2050 at 5%. Normalization is conducted with the 26by30 + 80by50_Def value being unity. The model median for each scenario is represented by a triangle

To assess the variability of each variable across models and scenarios, Fig. 10 presents the average carbon price discounted at 5%, normalized by its value for the 26by30 + 80by50_Def scenario. Based on the behavior of the medians (triangle in the diagram), stringent emissions constraints (90% and 100% reduction by 2050) are most impactful in increasing the costs, followed by non-availability of CCS and nuclear power. This is followed by sensitivity analyses on renewables and systems integration. Lower levels of demand can significantly reduce the costs, and the lowering of the industrial service demand reduces the cost substantially. Doubling the VRE potential and halving the VRE costs are also helpful in reducing the cost. High-energy import costs do not have a significant impact.

**Fig. 11:**
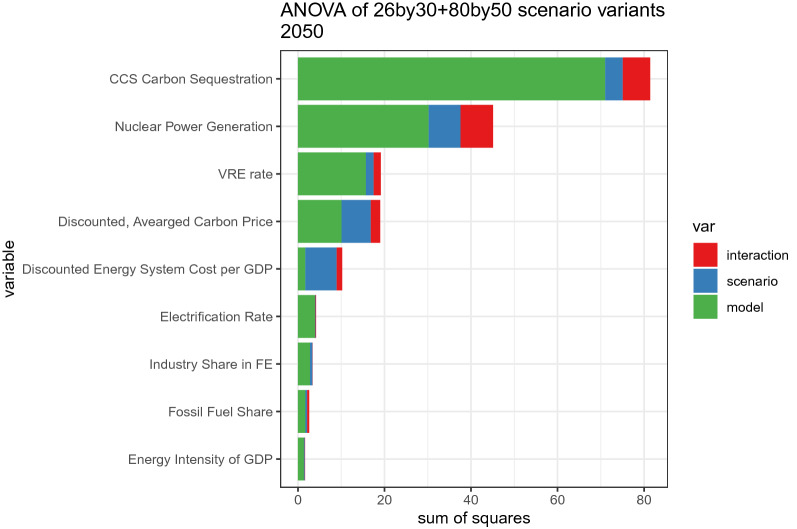
The sums of squares of the two-way ANOVA of each variable. The time period is 2050, except for cumulative variables. A discount rate of 5% is applied for discounted variables

An analysis based on a two-way ANOVA model reveals both uncertain metrics (e.g., costs, the role of nuclear, CCS, and VREs) and robust indicators (e.g., economy-wide energy efficiency, electrification). Figure 11 depicts the results of decomposition of the sum of squares of key variables, based on a two-way ANOVA model. Except for cost metrics, the variations are dominated by inter-model uncertainty, and inter-scenario variation plays a minor role. While CCS tops the list of the uncertainty among variables, all the cost metrics loom large because of model and scenario uncertainties. Both total and marginal cost metrics are sensitive, and scenario uncertainties are large, especially for the energy system cost. The shares of nuclear power and VREs are also susceptible to the choice of model and scenario. Besides reconfirming the findings of Figs. 5 and 11 clarifies where uncertainty prevails.

Note that the CO_2_ intensity of electricity is close to zero and has been excluded from this analysis.

## Discussion and conclusion

### Summary of modeling results

The present study has identified robust mitigation strategies that cut across models and scenarios. In spite of a diverse set of modeling frameworks, the models find economy-wide energy efficiency and electricity decarbonization to be the most robust. All the models find increasing trends of end-use electrification, deployment of VREs, and a shift away from fossil fuels, though the magnitudes vary among models. Partial equilibrium models also indicate that the residual emissions from the industry sector are difficult to abate. These are largely consistent with the literature and previous research (see the “Results”). Though not all models show feasible solutions for stringent policy scenarios (90% and 100% emissions reduction), the overall strategies remain the same and they are enhanced further.

Another robust feature is the stringency and coverage of future climate policy. The marginal cost or carbon price is set to increase rapidly. The 26by30 + 80by50_Def scenario shows a median price of ~ 70 2010USD/t-CO_2_ in 2030 and ~ 800 USD/t-CO_2_ in 2050, whereas the 26by30 + 80by50_LoDem scenario exhibits a median price of ~ 18 USD/t-CO_2_ in 2030 and ~ 709 USD/t-CO_2_ in 2050. Accordingly, policies must be strengthened to meet Japan’s NDC and mid-century strategy goals. All the emission sectors must contribute to mitigation with an exponentially rising marginal cost. In terms of the total cost, this translates into a 3% consumption loss per GDP for AIM/Hub-Japan and an additional total energy system cost of 0.8–0.9% of GDP for the partial equilibrium models in 2050.

These models also suggest areas of uncertainty. One such area is the energy mix. The models reveal multiple energy futures that are economically efficient. Another uncertain aspect is the exact size of the cost, which depends on both the model and scenario assumption. Both marginal and total costs vary greatly by model and assumptions, such as technology availability, service demand levels, and policy stringency.

### Policy implications

In the following, we provide the implications for policy based on our interpretation of modeling results.

The current mid-century strategy has not detailed any sectoral breakdown, and given the uncertainty in the industrial mitigation, policymakers should carefully design sectoral policies. On the other hand, power sector decarbonization is robust across models and scenarios. As discussed in the policy review section, the government has established a 2030 target, but not for 2050. The government should clarify the overall, 2050 power sector target in the future policy.

The current study reveals an exponential rise in carbon prices. As reviewed in policy review, currently, the carbon tax of Japan stands at ~ 3 USD/t-CO_2_. While the effective price is higher in some sectors, the current policy framework has not resulted in ambitious actions. Therefore, mitigation efforts need to be greatly expanded so that effective carbon pricing increases several-fold and covers all the sectors. The Organization for Economic Cooperation and Development (OECD 2018) reports that at the 30 EUR/t-CO_2_ level, there is a 69% coverage gap of market instruments. Though this is indicative only of market instruments, our findings hint that climate policy must be substantially strengthened in both breadth and depth.

In the real world, the government does not necessarily have to rely on explicit carbon pricing; it can invoke regulations, research and demonstration, tax credits, subsidies, and information campaigns as implicit carbon pricing, though extremely stringent policies could be politically infeasible. As our models suggest, there are robust strategies that can be pursued by Japan, including energy efficiency improvement, power sector decarbonization, electrification, and development of variable renewables. Although the government is making significant efforts, these efforts must be further accelerated by strengthening all the (effective) policy instruments, including energy efficiency standards, renewable energy auctions, and demonstration and diffusion of early-stage technologies.

As costs are dynamic, they should not be taken at their face value (Grubb et al. 2015; Nemet 2019). They can be considerably reduced by innovation. Given the scale of cost reduction required, however, broad innovation efforts must be markedly expanded. The first target should be VREs, as our analysis shows that halving the VRE costs does significantly reduce the costs. It is no brainer since other countries have successfully slashed the costs (IRENA 2019; Shiraki et al. 2021). Japan needs to follow suit. Another key consideration is the role of CCS and hydrogen. Models suggest that either CCS or hydrogen is required on a large scale, and yet technology development remains at the level of demonstration projects. The government needs to strengthen market creation policies for these new technologies.

Nurturing innovation at such a grandiose scale is a huge challenge because of the fundamental uncertainty in innovation and their interaction with other sources of uncertainty. Moreover, the role of the government in innovation is often indirect given the complexity of the national innovation system; see Nemet (2019) for the case of solar photovoltaics. As seen in Figs. 6 and 7, models show divergent pathways for Japan’s energy system. Except for VREs, the role of individual technologies cannot be ascertained. Therefore, policymakers will have to employ adaptive management in recognition of contemporaneous technology progress. For instance, the current government pays significant attention to hydrogen as a clean energy carrier. The Tokyo 2020 Olympic and Paralympic games that have been postponed (as of this writing) are going to feature hydrogen in the Olympic flame. The Tokyo Metropolitan Government is planning to introduce 50 fuel-cell buses (Tokyo Metropolitan Government 2020). The government has an ambitious goal to slash the cost of hydrogen by approximately one-third to 30 JPY/Nm^3^ by 2030s (Ministerial Council on Renewable Energy, Hydrogen and Related Issues 2017). Although these efforts are laudable, innovation targets are easy to miss; hydrogen may come but not at the desired time nor in the expected form. In fact, the energy mix presented in Figs. 6 and 7 does not show a significant role of hydrogen in 2030. Even in 2050, only two models (IEEJ_Japan 2017 and TIME-Japan) show some penetration. Policymakers should take into consideration the uncertainty of the future technology development.

In other words, the climate policy package must incorporate adaptive management as an essential element. In light of the updating mechanism under the Paris Agreement, the Government of Japan should take full advantage of the opportunity to address uncertainties. This approach is already embedded in the Strategic Energy Plan, which focuses on multi-track scenarios. The details are yet to be fleshed out. However, in the medium term, there appears to be less flexibility. For instance, the NDC essentially stipulates energy mix in the medium term. In the previous energy plans, the rule of nuclear power fluctuated greatly thanks to optimism, a nuclear disaster, and public perception, which affected the prospect of mitigation (Fig. 2). Our results demonstrate that there is no single energy future for Japan (Figs. 6, 7). Policymakers should embrace diverse possibilities for 2030 as well as 2050 by incorporating flexibility into the policy framework.

### Study limitations and future research agenda

Though this study covered multiple models and addressed many different sources of uncertainty, there are several limitations to the present study.

First, there is an acute need for further model development. The infeasibility of 90% emissions reduction in two models and 100% reduction in three models, and the carbon price levels exceeding the cost of carbon dioxide removal (Fuss et al. 2018), imply that models must incorporate options, such as BECCS. The sensitivity analysis suggests the important role of industrial decarbonization (for marginal costs) and renewables (for total costs), and further improvement on these fronts would be crucial (Ju et al. 2021; Shiraki et al. 2021). As there is a wide range reported in the literature (Matsuo et al. 2018), it would be illuminating to conduct an inter-comparison dedicated to renewables.

Second, in this paper, we have focused on the time horizon of 2050. The 2050 net-zero emissions target emphasizes 2050, but there is a need to analyze what happens after 2050. Thus, the model framework should be expanded. Some models already have this capability and conducted such an analysis (Kato and Kurosawa 2019, 2021). This is an important research issue in the next iteration.

Third, we did not include global models (Oshiro et al. 2019) or some notable models of Japan (Ozawa et al. 2021; Takeda and Arimura 2021). Most of the participating models are based on partial equilibrium concepts. The global models that include Japan as a distinct region do not necessarily represent Japan with the most up-to-date parameters. The Japanese research teams have advantages with data updating because of proximity and the language whereas global models have strengths in terms of comprehensiveness. Therefore, it is useful to compare global and national models in a more consistent manner. Although Oshiro et al. (2019) have considered only two models from Japan, their work is the first step in the right direction.

Fourth, we did not analyze all sources of uncertainties, nor did we analyze why models differ from each other. As of this writing, the COVID-19 pandemic crisis has had significant impacts on final energy and CO_2_ emissions as well as the possible future energy trajectories. All of our models and scenarios have missed it. More importantly, the effect of the base year should ideally be fully explored, but this aspect has not been analyzed. These issues are left for future research.

Fifth, the models did not represent any policy except for economy-wide carbon pricing. Some studies have begun work on this front (Roelfsema et al. 2020), and more realistic representation of policies would be crucial in the future.

## Supplementary Information

Below is the link to the electronic supplementary material.

Supplementary file1 (DOCX 1679 KB)

## Footnotes

1 The fiscal year runs from April 1st until March 31st of the following year.

2 No broad-based, stringent climate policy exists in Japan as of this writing.

3 Whether blast furnace gas is counted in the energy conversion sector or the industry sector makes a non-negligible difference. This difference affects both the emissions and final energy, and hence the changes reported in this paper.
