# Supplementary material for: EMF 35 JMIP study for Japan’s long-term climate and energy policy: scenario designs and key findings
Source: Sustain Sci. 2021 Feb 17;16(2):355–74. doi: 10.1007/s11625-021-00913-2 (PMC7887567; doi:10.1007/s11625-021-00913-2)
Supplement: Supplementary file 1 — Supplementary file1 (DOCX 1679 KB) [file 11625_2021_913_MOESM1_ESM.docx]

Electronic Supplementary Materials

# Model descriptions

## AIM/Enduse-Japan V2.1

AIM/Enduse-Japan is a partial equilibrium, dynamic recursive model which is characterized by the detailed descriptions of energy technologies in the end-use sectors as well as the energy supply sectors in Japan. This model is characterized by detailed representation of technologies, in which technologies are selected by linear programing minimizing total energy system costs given exogenous parameters such as energy service demands, energy prices, technological parameters, and carbon prices or emissions constraints. The model covers energy-related GHG emissions from both energy end-use and energy supply sectors. The end-use sectors are composed of industry, buildings and transportation sectors, and they are disaggregated into several subsectors with respect to types of products, buildings, and transportation mode based on the IEA energy balances. In the industry, building and transportation sectors, wide mitigation options are included, such as energy-efficient devices and fuel switching. This model covers not only energy sectors but also non-energy sectors such as industrial processes. It covers 10 sub-regions in Japan which is broadly coinciding with the areas of 10 public power supply firms, so as to consider characteristics of energy supply and demand across the various regions. The electricity dispatch module, that is hard linked with the energy end-use and other energy supply sectors module, explicitly represents the load curve in each region by 1-hour time steps, and capacity of electricity interconnection between sub-regions. The power sector also covers the measures to integrate VREs into the grid, such as electricity storage, demand response (DR) using battery-powered electric vehicles and heat pump devices, and interconnections. The diffusion rate of these technologies is estimated based on cost optimization.

Oshiro, K., Masui, T. (2015). Diffusion of low emission vehicles and their impact on CO2 emission reduction in Japan. *Energy Policy*, 81, 215-225.

Fujimori, S., Oshiro, K., Shiraki, H., Hasegawa, T. (2019). Energy transformation cost for the Japanese mid-century strategy. *Nature Communications*, 10(1), 4737.

Kainuma, M., Matsuoka, Y., Morita, T. (2003). *Climate policy assessment: Asia-Pacific integrated modeling*. Tokyo: Springer.

## AIM/Hub-Japan V2.1

AIM/Hub-Japan V2.1 is a single national model part of the Asia-Pacific Integrated Assessment Model (AIM) family of models, which is applied for assessing climate policies in Japan (Silva Herran et al., 2019). It derives from the AIM/Hub model, a one‐year‐step recursive‐type dynamic general equilibrium model that covers all regions of the world aggregated into 17 regions and including 42 industrial classifications. For appropriate assessment of bioenergy and land use competition, agricultural sectors are also highly disaggregated. Details of the model structure and mathematical formulae are described by Fujimori et al. (2017). The production sectors are assumed to maximize profits under multi‐nested constant elasticity substitution (CES) functions and each input price. Energy transformation sectors input energy and value added are fixed coefficients of output. They are treated in this manner to deal with energy conversion efficiency appropriately in the energy transformation sectors. Power generation values from several energy sources are combined with a Logit function. This functional form was used to ensure energy balance as the CES function does not guarantee an energy balance. Household expenditures on each commodity are described by a linear expenditure system function. The parameters adopted in the linear expenditure system function are recursively updated in accordance with income elasticity assumptions. In addition to energy‐related CO_2_, the model includes CO_2_ from other sources, CH_4_, N_2_O, and fluorinated gases (F‐gases). Energy‐related emissions are associated with fossil fuel feedstock use. The non‐energy‐related CO_2_ emissions consist of land use change and industrial processes. Land use change emissions are derived from the forest area change relative to the previous year multiplied by the carbon stock density, which is differentiated by AEZs (Global Agro‐Ecological Zones). Non‐energy‐related emissions other than land use change emissions are assumed to be in proportion to the level of each activity (such as output). CH_4_ has a range of sources, mainly the rice production, livestock, fossil fuel mining, and waste management sectors. N_2_O is emitted as a result of fertilizer application and livestock manure management, and by the chemical industry. F‐gases are emitted mainly from refrigerants used in air conditioners and cooling devices in industry. Air pollutant gases (BC, CO, NH_3_, NMVOC, NOX, OC, SO_2_) are also associated with fuel combustion and activity levels. Essentially, emissions factors change over time with the implementation of air pollutant removal technologies and relevant legislation.

Fujimori S., Hasegawa S., Masui T. (2017) AIM/CGE V2.0: Basic Feature of the Model. In: Fujimori S., Kainuma M., Masui T., Post‐2020 Climate Action: Global and Asian Perspective, Springer, 305‐328

Fujimori S., Masui T., Matsuoka Y. (2017) AIM/CGE V2.0 Model Formula. In: Fujimori S., Kainuma M., Masui T., Post‐2020 Climate Action: Global and Asian Perspective, Springer, 201‐303

Fujimori S., Hasegawa T., Masui T., Takahashi K., Silva Herran D., Dai H., Hijioka Y., Kainuma M. (2017) SSP3: AIM implementation of Shared Socioeconomic Pathways. Global Environmental Change, 42, 268‐283

Silva Herran D., Fujimori S., Kainuma M. (2019) Implications of Japan’s long term climate mitigation target and the relevance of uncertain nuclear policy. Climate Policy, 19:9, 1117-1131.

## DNE21 V1.3

Dynamic New Earth 21 model (DNE21) is an integrated assessment model that provides a framework for evaluating the optimal global energy mix to stabilize low atmospheric CO_2_ concentrations (Hosoya and Fujii, 2011; Fujii and Komiyama, 2015). The model seeks the optimal solution that minimizes the total system cost, in multiple time stages for the years to 2100 at ten-year intervals in multiple regions, under various kinds of constraints, such as amount of resource, energy supply and demand balance, and CO_2_ emissions. The model is formulated as a linear optimization model. In the DNE21 model, the world is divided into 54 regions. In the model, large countries such as the United States, Russia, China, and India are further divided into several sub-regions. Furthermore, in order to reflect the geographical distribution of the site of regional energy demand and energy resource production, each region consists of “city nodes” and “production nodes”, the total number of which amounts to 82 points. The model takes detailed account of intra-regional and inter-regional transportation of fuel, electricity, and CO_2_ between these points. DNE21 involves various components that model energy production, conversion and transport, primary energy resources, secondary energy carriers, final energy demand sector, power generation technology, energy conversion process, and CO_2_ capture (3 types) and storage. End-use electricity demand is assumed with a specific daily electricity load curve divided into six time intervals. Additionally, DNE21 incorporates a nuclear module, which describes in detail the nuclear fuel cycle and nuclear technology such as light-water reactors (LWR), light-water mixed oxide fuel reactors (LWR-MOX), and fast breeder reactors (FBR). Furthermore, a photovoltaic power (PV) module was incorporated in the most recent version of the model. The intermittent characteristics of PV power generation due to changes in weather conditions are taken into account by stochastic programming.

Hosoya, Y., Fujii, Y., Analysis of energy strategies to halve CO_2_ emissions by the year 2050 with a regionally disaggregated world energy model, Energy Procedia, 43, pp.5853-5860, 2011

Fujii, Y., Komiyama, R., Long-term energy and environmental strategies, Ahn, J., Carson, C., Jensen, M., Juraku, K., Nagasaki, S., Tanaka, S.(Eds.), Reflections on the Fukushima Daiichi nuclear accident, Springer International Publishing, pp. 105-115, 2015

## IEEJ Japan model ver. 2017

IEEJ Japan model ver. 2017 was developed by the Institute of Energy Economics, Japan (Matsuo et al, 2013; Matsuo et al., 2020) to harmonize macroeconomic and energy technology analyses. It consists of three soft-linked submodels: a macroeconomic model, an optimal power generation mix model and an energy technology optimization model. The macroeconomic model is a one-regional Keynesian econometric model, which calculates various macroeconomic indicators and economic activities up to 2050, with assumptions of population growth, primary energy prices, and other indexes. Although gross domestic product (GDP) and its components are outputs from the model, it is also possible to fix future GDP growth as an assumption for calculation. The optimal power generation mix model is a linear programming optimization model, which calculates the cost-optimal power sector planning and operation, simulating temporal changes in electric loads and power outputs of variable renewable energies (VREs) with an hourly resolution. It divides Japan into nine regions, and considers the costs of interregional transmission lines, as well as of power storage systems including pumped hydro, batteries, and hydrogen storage. This model is used for calculating marginal integration costs of power sources with high shares of VREs (Matsuo and Komiyama, 2020). The energy technology optimization model is a one-regional MARKAL (MARket ALlocation)-type optimization model, which calculates the optimal deployment of energy-related technologies, using input data from other submodels (i.e. the economic activities calculated by the macroeconomic model, and the marginal integration costs calculated by the optimal power generation mix model). It also calculates final energy demand by sector and by energy source, primary energy demand by energy source, power generation mix, and energy-related CO_2_ emissions up to the year 2050. Although the model can consider future new construction of nuclear power plants, it only assumes the use of the currently existing nuclear plants with a lifetime of 60 years, for the calculations shown in this article.

Matsuo Y., Yanagisawa A., Yamashita Y. (2013) A global energy outlook to 2035 with strategic considerations for Asia and Middle East energy supply and demand interdependencies. Energy Strategy Reviews, 2, 79-91.

Matsuo Y., Endo S., Nagatomi Y., Shibata Y., Komiyama R., Fujii Y. (2020) Investigating the economics of the power sector under high penetration of variable renewable energies. Applied Energy, 267, 113956.

Matsuo Y., Komiyama R. (2020) System LCOE of variable renewable energies: A case study of Japan’s long-term GHG mitigation target towards 2050. Sustainability Science (this issue)

## TIMES-Japan 3.1

TIMES-Japan is a regional model developed at the Institute of Applied Energy (Kurosawa and Hagiwara 2011; Kato and Kurosawa 2019) using the TIMES modeling framework developed by IAE ETSAP (Loulou et al. 2005). The model consists of Japan’s detailed energy systems based on the energy flow and parameters of MARKAL-JAPAN (Sato 2005), with an additional energy carrier (imported hydrogen) incorporated. The model follows a whole system approach, conducting least-cost optimization calculations under exogenous service-demand assumptions. The objective function is discounted sum of total energy system cost with a discount rate of 3% per year. The reference energy system in TIMES-Japan used in this study consists of 399 processes and 163 commodities. In each set of calculations, an optimized combination of renewable and non-renewable energy sources on the supply side meet the final energy service demands for industrial productions, transportations and buildings. The CO_2_ emissions from energy sectors and industrial processes are accounted in the framework.

Kato E, Kurosawa A (2019) Evaluation of Japanese energy system toward 2050 with TIMES-Japan – deep decarbonization pathways. Energy Procedia 158, 4141–4146

Kurosawa A, Hagiwara N (2012) Long term energy system analysis of Japan after March 11, 2011, 3rd IAEE Asian Conference, February 21, 2012: https://eneken.ieej.or.jp/3rd_IAEE_Asia/pdf/paper/106p.pdf [accessed April 29, 2020]

Loulou R, Remne U, Kanudia A, Lehtila A, Goldstein G (2005) Documentation for the TIMES model - part I. http://iea-etsap.org/docs/TIMESDoc-Intro.pdf [accessed April 29, 2020]

Sato O (2005) A study on long-term energy scenarios for Japan. JAERI-Research 2005-012: http://doi.org/10.11484/jaeri-research-2005-012 (in Japanese) [accessed April 29, 2020]

# Scenario descriptions

As described in the text, our scenario is denoted in the format of *(policy dimension)_(other parameter settings)*.

## Policy dimension

For models with land use and non-CO_2_ emissions, modeling teams should apply the same carbon price across energy CO_2_, land use CO_2_, and non-CO_2_ greenhouse gases. Emission pathways should be linear in time.

- **Baseline**: A baseline scenario. Left to the individual modeling group's choice.
- **26by30+80by50**: A mitigation scenario, which combines the FY2030 NDC goal (26% reduction relative to FY2013 levels) and the 2050 goal (80% reduction).
- **26by30+70by50**: As in 26by30+80by50 except for the 2050 goal is replaced with 70% reduction.
- **26by30+90by50**: As in 26by30+80by50 except for the 2050 goal is replaced with 90% reduction.
- **26by30+100by50**: As in 26by30+80by50 except for the 2050 goal is replaced with 100% reduction.
- **16by30+80by50**: As in 26by30+80by50 but with the 2030 reduction levels at 16%.
- **36by30+80by50**: As in 26by30+80by50 but with the 2030 reduction levels at 36%.

## Other parameter settings

### Supply-side Technology dimension

- **Def**: A default set of technology assumptions, which are left to modelers.
- **NoCCS**: CCS deployment should be zero for the entire period.
- **LimNuc**: Nuclear deployment should be set as follows (and linearly interpolated)
  - 2015=actual level, 2030=NDC,
    2050=25.5 GW = 178.704 TWh (assuming 60-year lifetime extension, 3 new builds, and an 80% capacity factor)
- **NoNuc**: 2050 nuclear deployment = 0 since 2011
- **HighInt:**  Stationary battery costs should be set to the larger of the two: the model default value in 2015 or $600/kWh. No VRE curtailment should be allowed. Measures other than batteries and curtailment (e.g., hydrogen conversion and demand response) should not be changed.
- **LowInt:** Stationary battery costs should be drastically reduced to $50/kWh in 2050 according to a linear schedule from the default value in 2015. VRE curtailment should be allowed for models with a curtailment option in default.
- **LowVREcost**: Capital costs for wind and solar halved^^[[1]](#footnote-1)^^.
- **HighVREcos**t: Capital costs for wind and solar doubled.
- **LowVREpot**: Total resource potential for wind and solar halved for 2020 and onwards. For the model assumed multiple grade (different capacity factors) for VRE, potentials for each resource grade should be halved.
- **HighVREpot**: Total resource potential for wind and solar doubled for 2020 and onwards. For the model assumed multiple grade (different capacity factors) for VRE, potentials for each resource grade should be doubled.
- **LoStorageCost:** Stationary battery costs should be drastically reduced to $1/kWh in 2050 according to a linear schedule from the default value in 2015. VRE curtailment should be allowed for models with a curtailment option in default.

### Demand

- **LoDem**: SSP2 per-capita GDP growth throughout
- **LoDemInd**: SSP2 per-capita GDP growth throughout. On top of it, the industrial energy service demand should be reduced by 0% in the model starting year, 50% in 2030, and 50% in 2050, compared to the levels found in the LoDem scenario. Linear interpolation should be used for the time periods in between.
- **LoDemTra**: The same as above but for the transportation sector.
- **LoDemBld:** The same as above but for the buildings sector (=residential & commercial).

### Import

- **HiIm**: Prices of clean energy imports doubled. Clean energy here includes hydrogen, biomass, and electricity (if international grid interconnection is considered). Note that if noted indicated, default assumptions on clean energy imports apply.

## Harmonization of other input parameters

### GDP

We employ two GDP scenarios.

In the reference GDP scenario, we use the same GDP project as was used for *the Long-term Energy Demand and Supply Outlook*  (METI 2015)^^[[2]](#footnote-2)^^ through 2030, and extend that with SSP2 growth rate (IIASA SSP database) (in particular, the OECD GDP growth rate (Dellink 2017))^^[[3]](#footnote-3)^^.

For an alternative GDP scenario, we use the growth rates from SSP2 throughout.

For general equilibrium models with endogenous economic growth, it is expected that the models will adjust the growth rate to reflect the specified growth rate to the extent possible.

The 2015 long-term energy outlook^^[[4]](#footnote-4)^^ refers to the financial outlook by the government^^[[5]](#footnote-5)^^. This financial outlook provides the annual breakdown of growth rates, but for the purpose of EMF 35 JMIP, the modeling team is encouraged to use a constant growth rate of 1.7%/yr for the period up to 2030.

### Table ESM 1. Reference GDP scenario.

| Year | Annual growth rate |
| --- | --- |
| Until 2030 | 1.700% |
| 2030-2040 | 0.583% |
| 2040-2050 | 0.522% |

### Table ESM 2. SSP2 GDP scenario.

| Year | Annual growth rate |
| --- | --- |
| 2010-2020 | 1.032 % |
| 2020-2030 | 0.982% |
| 2030-2040 | 0.583% |
| 2040-2050 | 0.522% |

### Population

We use the middle population projection by the National Institute of Population and Social Security Research (IPSS, 2017)^^[[6]](#footnote-6)^^. The following table describes numerical values.

### Table ESM 3. Population projection by IPSS (2017).

| Year | Population in thousands |
| --- | --- |
| 2015 | 127,095 |
| 2016 | 126,838 |
| 2017 | 126,532 |
| 2018 | 126,177 |
| 2019 | 125,773 |
| 2020 | 125,325 |
| 2021 | 124,836 |
| 2022 | 124,310 |
| 2023 | 123,751 |
| 2024 | 123,161 |
| 2025 | 122,544 |
| 2026 | 121,903 |
| 2027 | 121,240 |
| 2028 | 120,555 |
| 2029 | 119,850 |
| 2030 | 119,125 |
| 2031 | 118,380 |
| 2032 | 117,616 |
| 2033 | 116,833 |
| 2034 | 116,033 |
| 2035 | 115,216 |
| 2036 | 114,383 |
| 2037 | 113,535 |
| 2038 | 112,674 |
| 2039 | 111,801 |
| 2040 | 110,919 |
| 2041 | 110,028 |
| 2042 | 109,131 |
| 2043 | 108,229 |
| 2044 | 107,326 |
| 2045 | 106,421 |
| 2046 | 105,518 |
| 2047 | 104,616 |
| 2048 | 103,716 |
| 2049 | 102,819 |
| 2050 | 101,923 |

## Scenario submission status

The following table summarizes the status of scenario submissions.

### Table ESM 4. Status of scenario submissions. (1=submission.)

|  | AIM/Enduse[Japan] | AIM/Hub-Japan | DNE21 | IEEJ_Japan 2017 | TIMES-Japan |
| --- | --- | --- | --- | --- | --- |
| 16by30+80by50_Def | 1 | 1 | 1 | 1 | 1 |
| 26by30+100by50_Def |  | 1 | 1 |  |  |
| 26by30+70by50_Def | 1 | 1 | 1 | 1 | 1 |
| 26by30+80by50_Def | 1 | 1 | 1 | 1 | 1 |
| 26by30+80by50_HighInt | 1 | 1 | 1 | 1 | 1 |
| 26by30+80by50_HiImportCost | 1 | 1 | 1 | 1 | 1 |
| 26by30+80by50_HiVREcost | 1 | 1 | 1 | 1 | 1 |
| 26by30+80by50_HiVREpot | 1 | 1 | 1 | 1 | 1 |
| 26by30+80by50_LimNuc | 1 | 1 | 1 | 1 | 1 |
| 26by30+80by50_LoDem | 1 | 1 | 1 | 1 | 1 |
| 26by30+80by50_LoDemBld | 1 | 1 |  | 1 | 1 |
| 26by30+80by50_LoDemInd | 1 | 1 |  | 1 | 1 |
| 26by30+80by50_LoDemTra | 1 | 1 |  | 1 | 1 |
| 26by30+80by50_LoInt | 1 | 1 | 1 | 1 | 1 |
| 26by30+80by50_LoStorageCost | 1 | 1 | 1 | 1 | 1 |
| 26by30+80by50_LoVREcost | 1 | 1 | 1 | 1 | 1 |
| 26by30+80by50_LoVREpot | 1 | 1 | 1 | 1 | 1 |
| 26by30+80by50_NoCCS | 1 | 1 | 1 | 1 | 1 |
| 26by30+80by50_NoNuc | 1 | 1 | 1 | 1 | 1 |
| 26by30+90by50_Def | 1 | 1 | 1 |  |  |
| 36by30+80by50_Def | 1 | 1 | 1 | 1 | 1 |
| Baseline_Def | 1 | 1 | 1 | 1 | 1 |
| Baseline_HighInt | 1 | 1 | 1 | 1 | 1 |
| Baseline_HiImportCost | 1 | 1 | 1 | 1 | 1 |
| Baseline_HiVREcost | 1 | 1 | 1 | 1 | 1 |
| Baseline_HiVREpot | 1 | 1 | 1 | 1 | 1 |
| Baseline_LimNuc | 1 | 1 | 1 | 1 | 1 |
| Baseline_LoDem | 1 | 1 | 1 | 1 | 1 |
| Baseline_LoDemBld | 1 | 1 |  | 1 | 1 |
| Baseline_LoDemInd | 1 | 1 |  | 1 | 1 |
| Baseline_LoDemTra | 1 | 1 |  | 1 | 1 |
| Baseline_LoInt | 1 | 1 | 1 | 1 | 1 |
| Baseline_LoStorageCost | 1 | 1 | 1 | 1 | 1 |
| Baseline_LoVREcost | 1 | 1 | 1 | 1 | 1 |
| Baseline_LoVREpot | 1 | 1 | 1 | 1 | 1 |
| Baseline_NoCCS | 1 | 1 | 1 | 1 | 1 |
| Baseline_NoNuc | 1 | 1 | 1 | 1 | 1 |

# Energy data sources and model treatment

The models differ in their representation of the industry sector, and the energy allocation does not strictly follow that of the energy balance. For instance, AIM/Hub-Japan and AIM/Enduse-Japan are both based on the IEA energy balance. The former represents the blast furnace in the transformation sector whereas AIM/Enduse-Japan incorporates it in the industry end-use sector. As an illustration, the IEA extended world energy balance shows a total final consumption of 14EJ/yr, an industry final energy consumption of 4.3EJ/yr, a non-energy use of 1.5EJ/yr, and a transformation loss in the blast furnace of 0.77EJ/yr, all for 2005. A simple calculation suggests that a slight change in the allocation does affect the share of industry in final energy. For instance, (industry + non-energy use) / (total final energy) = 41.8%, which is close to the AIM/Hub-Japan value, whereas (industry + non-energy use + blast furnace) / (total final energy + blast furnace) = 44.8%, which is close to the AIM/Enduse-Japan.

Table ESM 4. Data sources of energy balances and emissions.

|  | AIM/Hub-Japan | AIM/Enduse-Japan | IEEJ_Japan | TIMES-Japan |
| --- | --- | --- | --- | --- |
| Energy balance data source | IEA | IEA | IEA | EDMC statistics |
| Emissions data source | EDGAR | National GHG Inventory Report | National GHG Inventory Report | National GHG Inventory Report |

#

# Additional figures

###
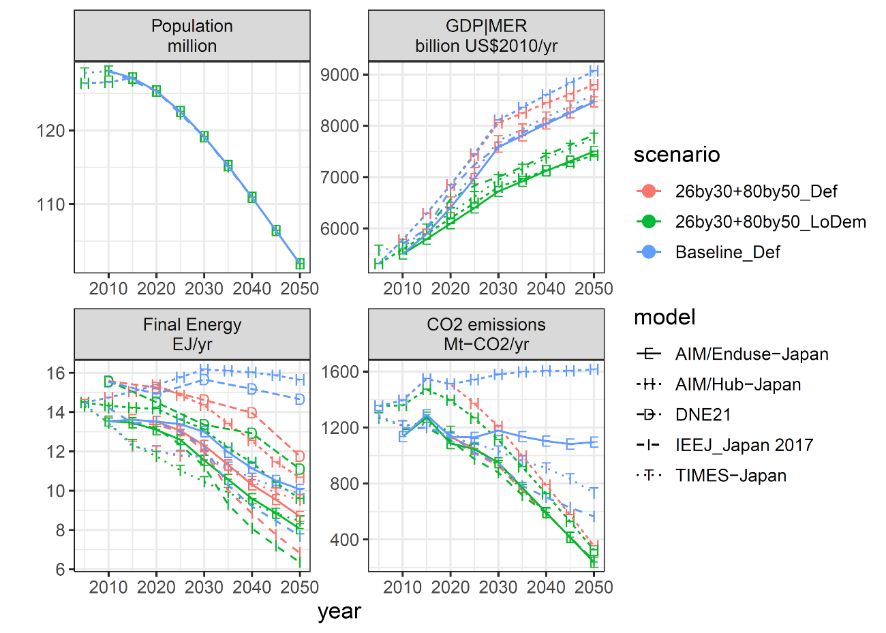
Fig. ESM 1. As in Fig. 3 but with additional points for the 26by30+80by50_LoDem scenario.


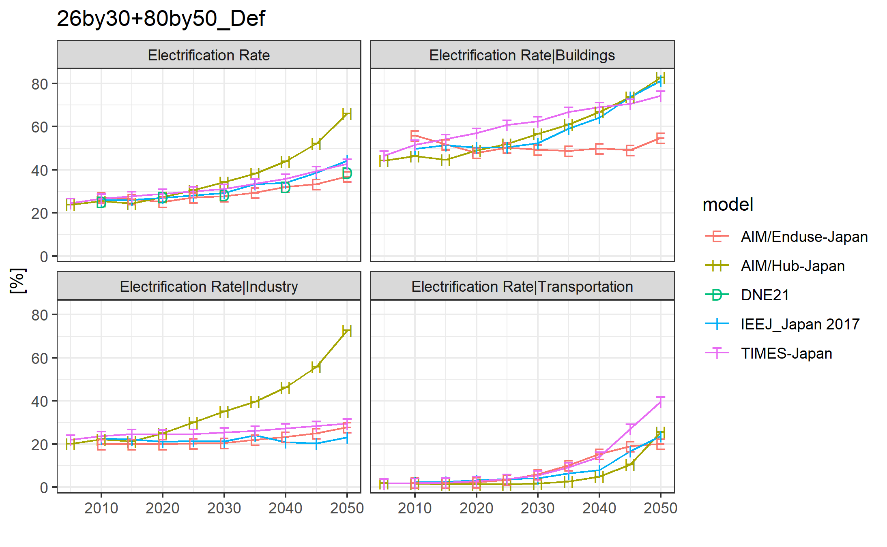


### Fig. ESM 2. Electrification rate for the 26by30+80by50_Def scenario by sector.


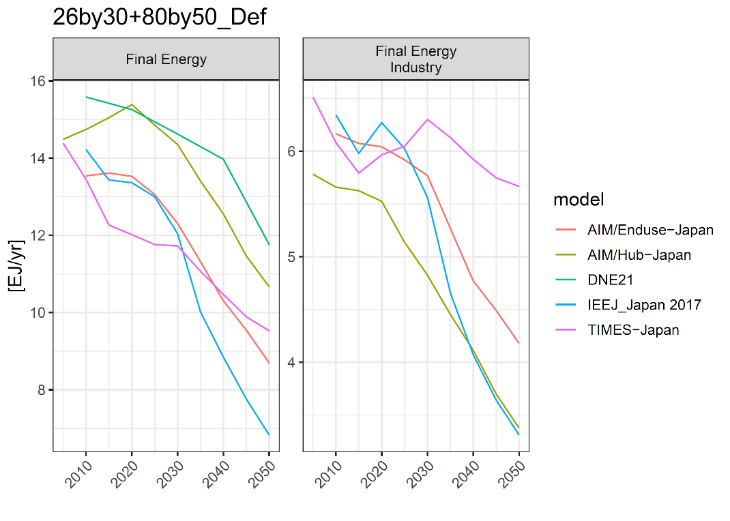


Fig. ESM 3. Total final energy consumption and industry final energy for the 26by30+80by50_Def scenario.


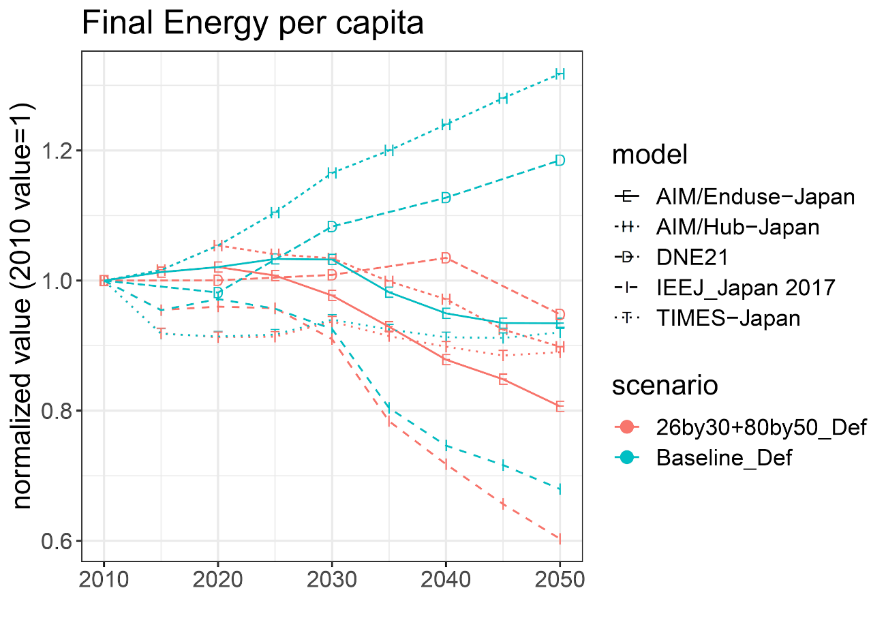


### Fig. ESM 4. Per-capita final energy consumption. Normalized so that the 2010 value = 1.


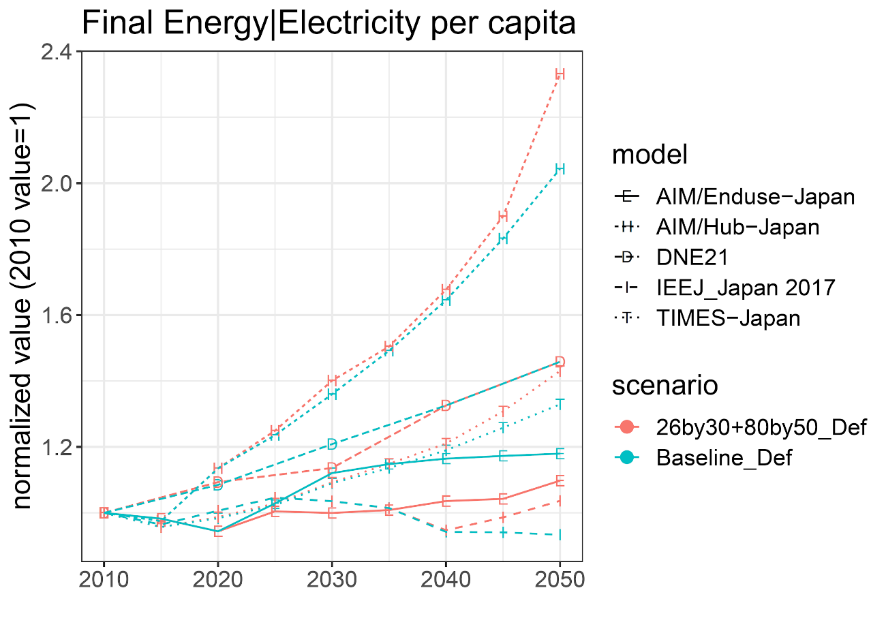


### Fig. ESM 5. Per-capita final energy consumption of electricity. Normalized so that the 2010 value = 1.

###

###
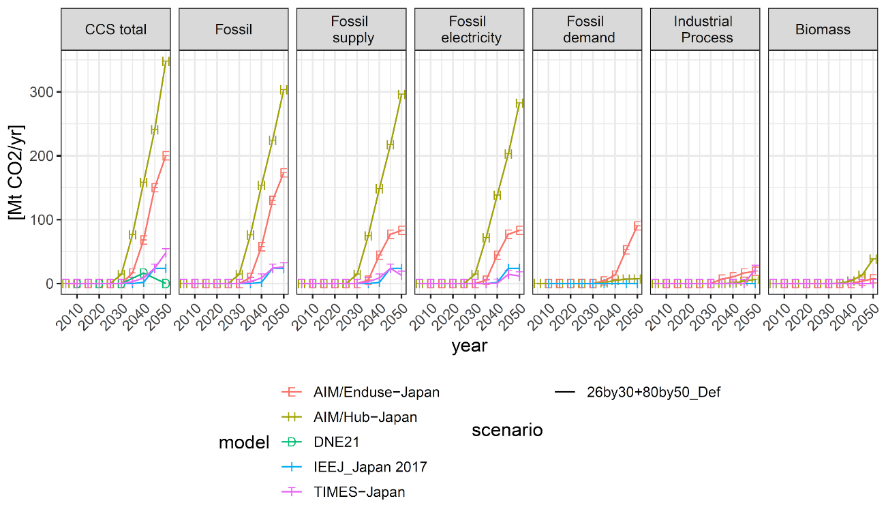
Fig. ESM 6. Decomposition of CCS carbon sequestration.

###

###

###
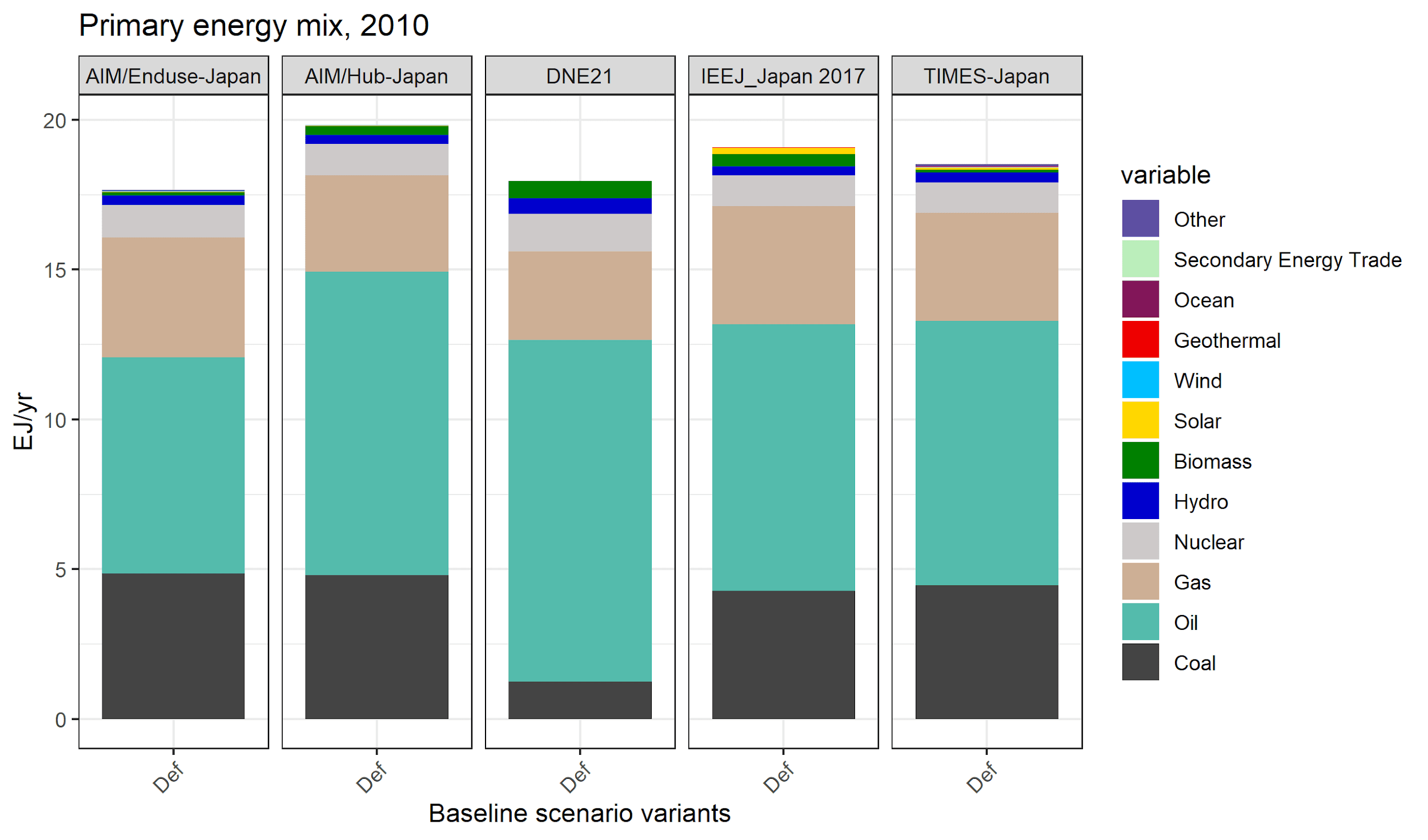

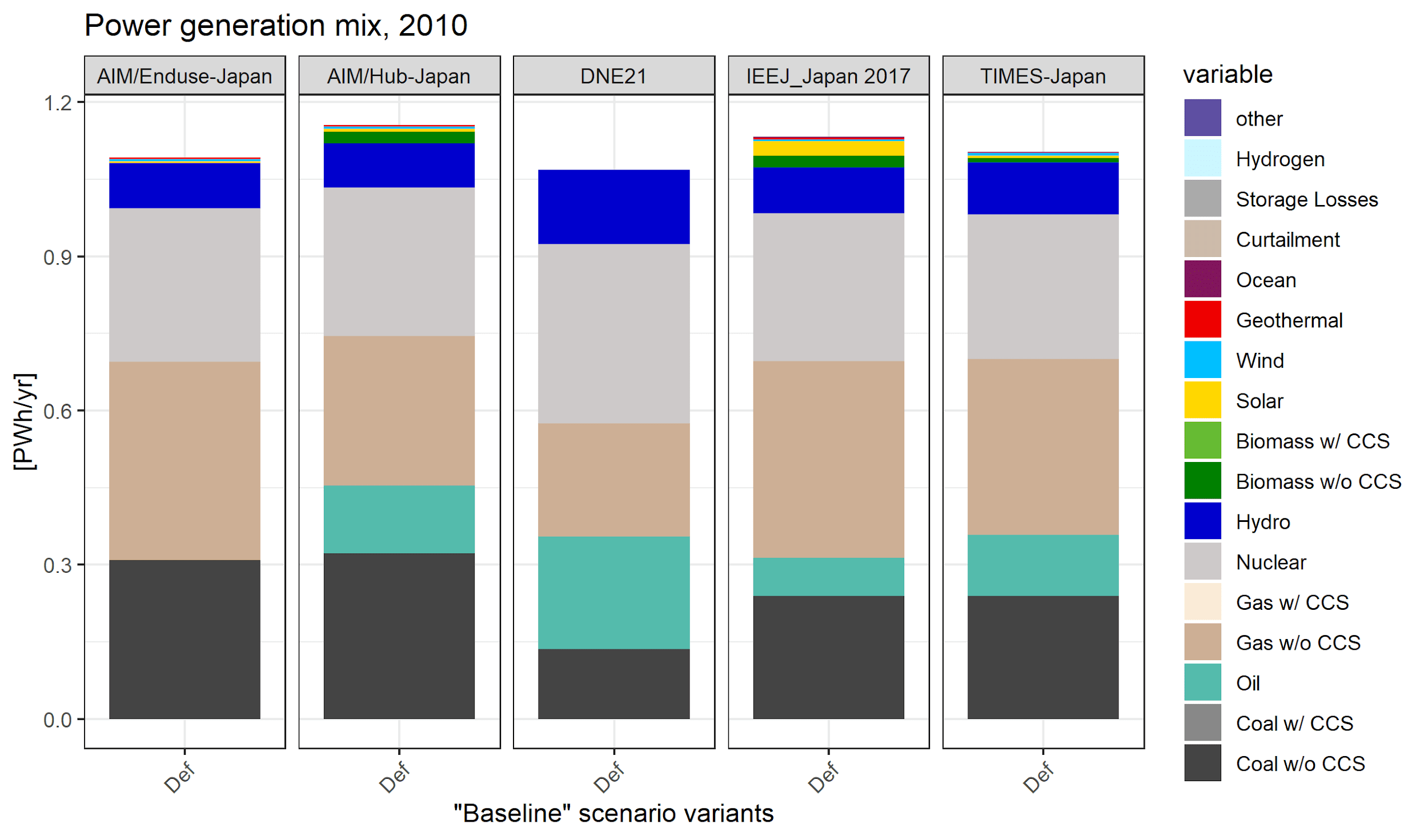
Fig. ESM 7. 2010 primary energy and electricity generation mixes.

###
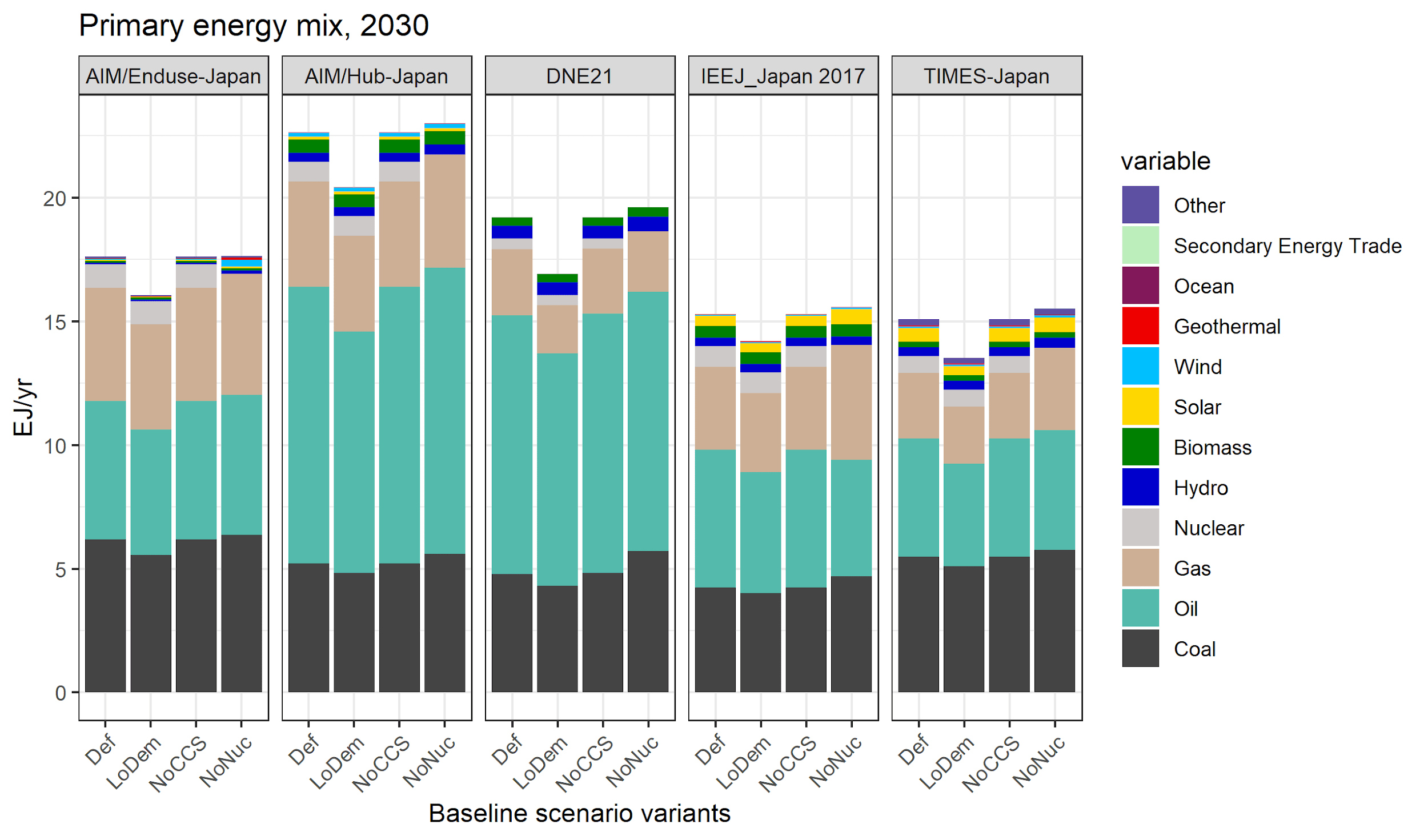


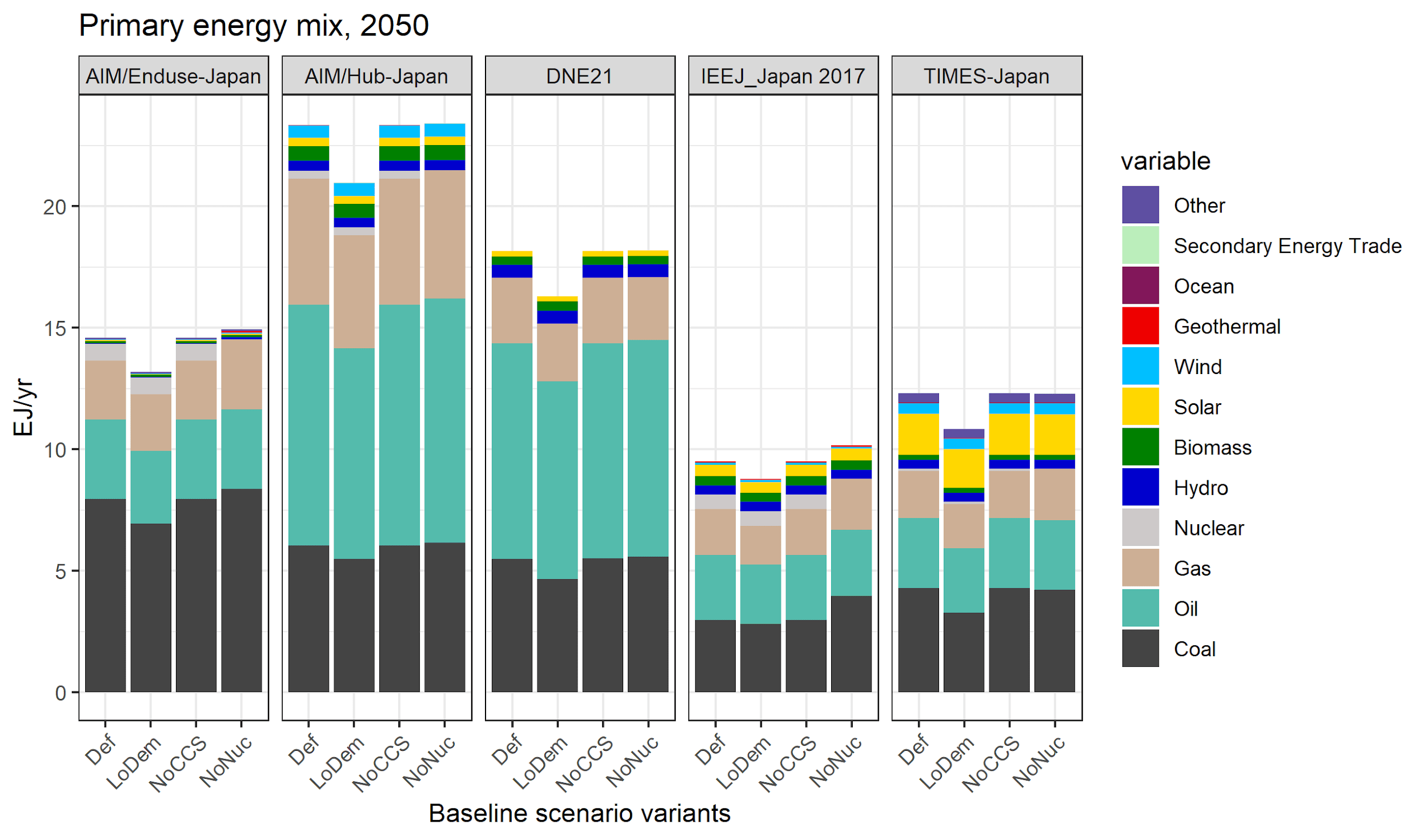


### Fig. ESM 8. Primary energy mix in the baseline in 2030 and 2050.

###


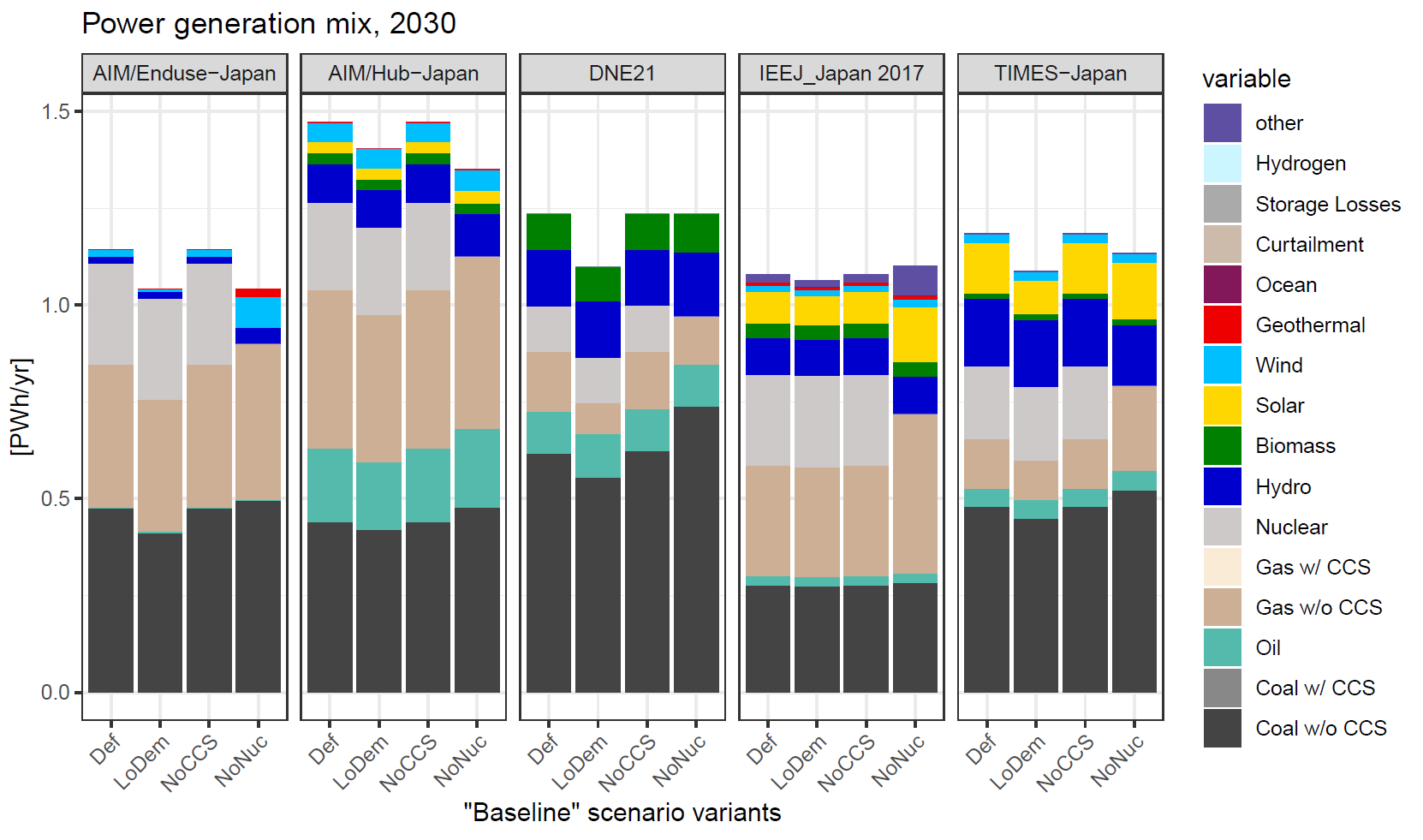


###


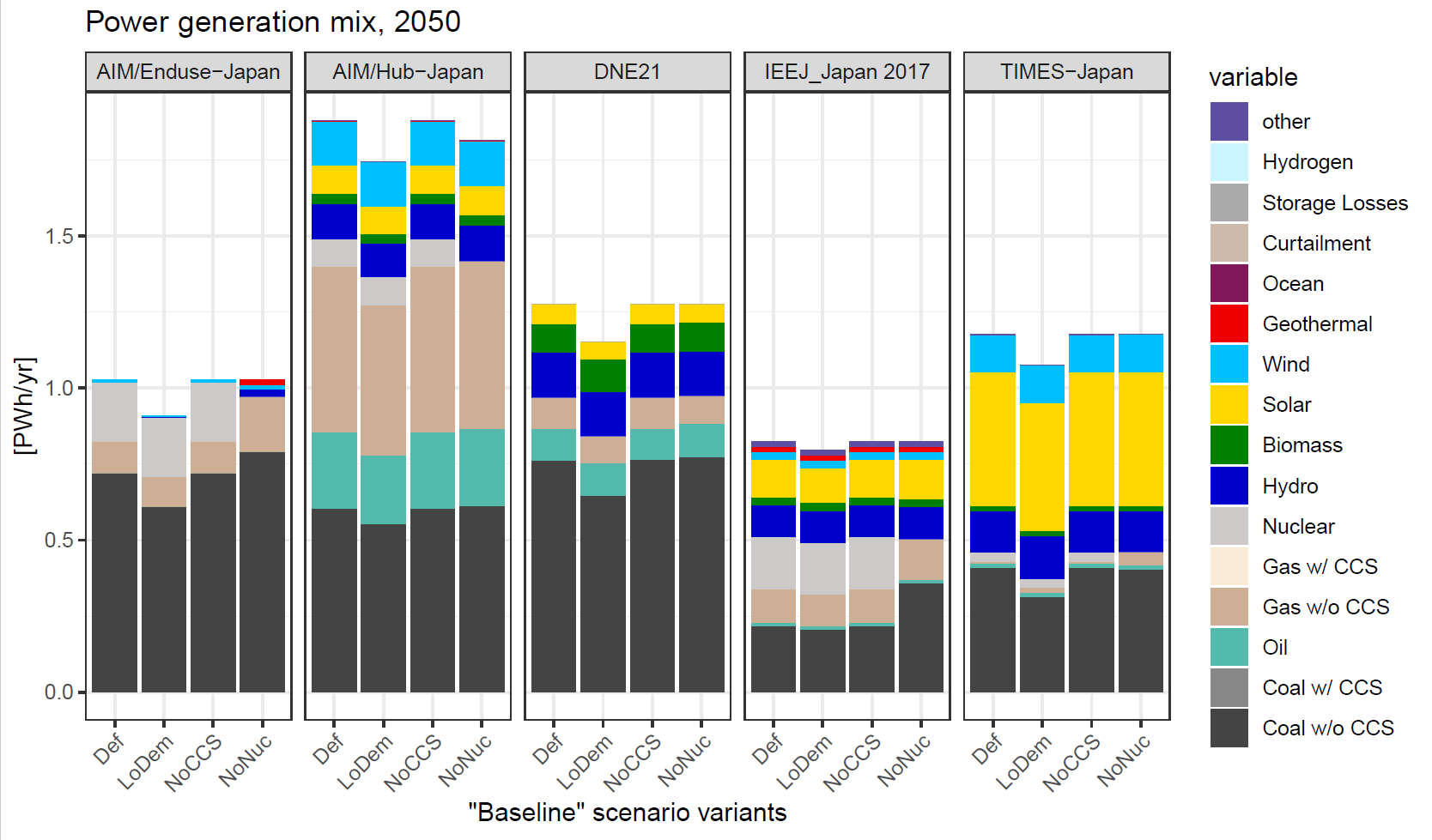


### Fig. ESM 9. Power generation mix in the baseline scenario for 2030 and 2050.


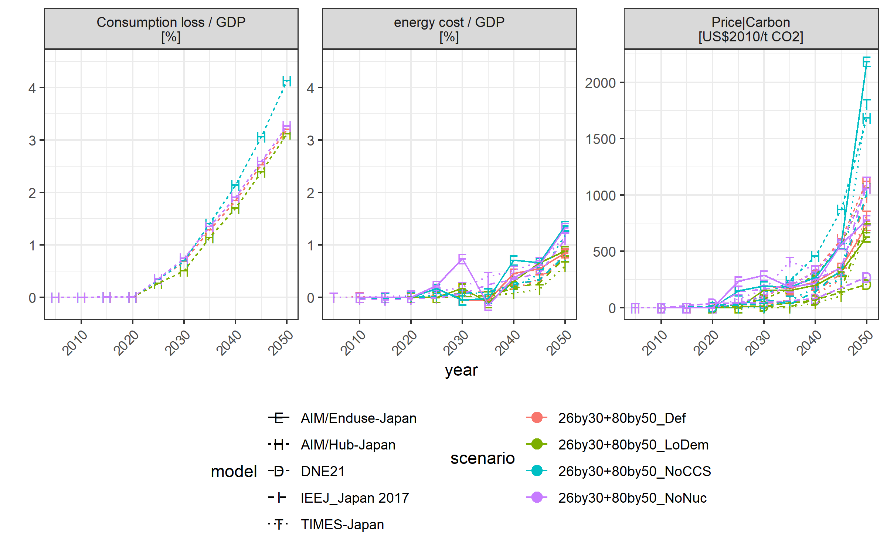


### Fig. ESM 10. Costs of individual scenarios.

###


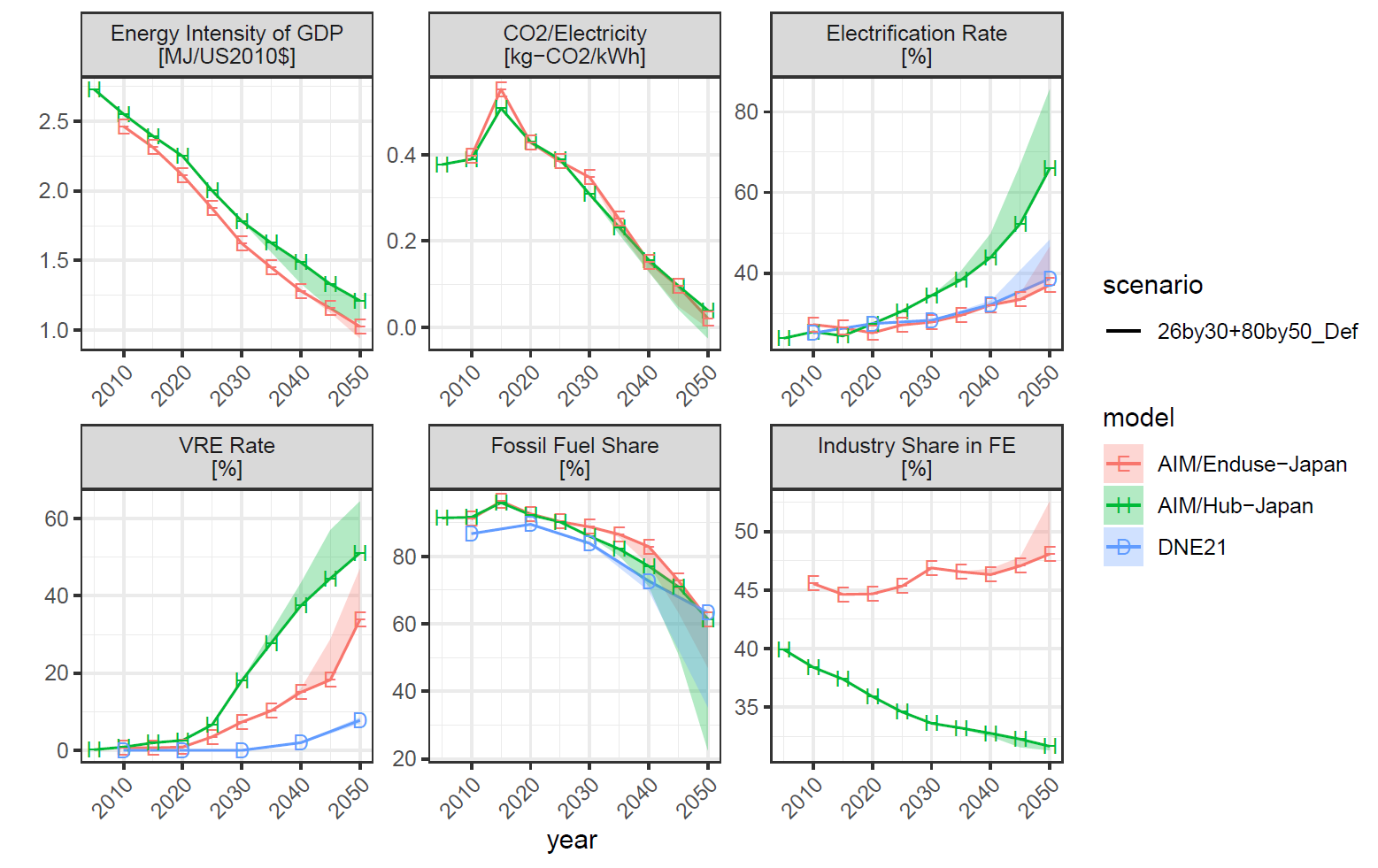


### Fig. ESM 11. Key indicators of decarbonization options as in Fig. 5 but for different levels of the climate policy stringency. The line represents the results for the 26by30+80by50_Def scenario whereas the ribbons represent the range of the 26by30+80by50_Def, 26by30+90by50_Def, 26by30+100by50_Def scenarios.

1. The timing of the cost change was not harmonized, which led to an underestimate of the effect of VRE cost reduction. [↑](#footnote-ref-1)
2. <http://www.meti.go.jp/press/2015/07/20150716004/20150716004_2.pdf> (footnote 3, page 4) [↑](#footnote-ref-2)
3. <https://tntcat.iiasa.ac.at/SspDb/dsd?Action=htmlpage&page=countries> [↑](#footnote-ref-3)
4. <https://www.enecho.meti.go.jp/committee/council/basic_policy_subcommittee/mitoshi/pdf/report_01.pdf> [↑](#footnote-ref-4)
5. <https://www5.cao.go.jp/keizai3/econome/h27chuuchouki2.pdf> [↑](#footnote-ref-5)
6. <http://www.ipss.go.jp/pp-zenkoku/j/zenkoku2017/pp29_gaiyou.pdf>

   An Excel sheet can be downloaded from here: <http://www.ipss.go.jp/pp-zenkoku/j/zenkoku2017/db_zenkoku2017/db_zenkoku2017gaiyo.html> [↑](#footnote-ref-6)
